# A mechanism for epithelial-mesenchymal heterogeneity in a population of cancer cells

**DOI:** 10.1371/journal.pcbi.1007619

**Published:** 2020-02-10

**Authors:** Shubham Tripathi, Priyanka Chakraborty, Herbert Levine, Mohit Kumar Jolly

**Affiliations:** 1 PhD Program in Systems, Synthetic, and Physical Biology, Rice University, Houston, TX, United States of America; 2 Center for Theoretical Biological Physics, Rice University, Houston, TX, United States of America; 3 Department of Physics, Northeastern University, Boston, MA, United States of America; 4 Centre for BioSystems Science and Engineering, Indian Institute of Science, Bangalore, Karnataka, India; Virginia Tech, UNITED STATES

## Abstract

Epithelial-mesenchymal heterogeneity implies that cells within the same tumor can exhibit different phenotypes—epithelial, mesenchymal, or one or more hybrid epithelial-mesenchymal phenotypes. This behavior has been reported across cancer types, both *in vitro* and *in vivo*, and implicated in multiple processes associated with metastatic aggressiveness including immune evasion, collective dissemination of tumor cells, and emergence of cancer cell subpopulations with stem cell-like properties. However, the ability of a population of cancer cells to generate, maintain, and propagate this heterogeneity has remained a mystifying feature. Here, we used a computational modeling approach to show that epithelial-mesenchymal heterogeneity can emerge from the noise in the partitioning of biomolecules (such as RNAs and proteins) among daughter cells during the division of a cancer cell. Our model captures the experimentally observed temporal changes in the fractions of different phenotypes in a population of murine prostate cancer cells, and describes the hysteresis in the population-level dynamics of epithelial-mesenchymal plasticity. The model is further able to predict how factors known to promote a hybrid epithelial-mesenchymal phenotype can alter the phenotypic composition of a population. Finally, we used the model to probe the implications of phenotypic heterogeneity and plasticity for different therapeutic regimens and found that co-targeting of epithelial and mesenchymal cells is likely to be the most effective strategy for restricting tumor growth. By connecting the dynamics of an intracellular circuit to the phenotypic composition of a population, our study serves as a first step towards understanding the generation and maintenance of non-genetic heterogeneity in a population of cancer cells, and towards the therapeutic targeting of phenotypic heterogeneity and plasticity in cancer cell populations.

## Author summary

Epithelial-mesenchymal heterogeneity, wherein cells within the same tumor can exhibit an epithelial, a mesenchymal, or one or more hybrid epithelial-mesenchymal phenotype(s), has been observed across cancer types and implicated in metastatic aggressiveness. Here, we have used computational modeling to show that this heterogeneity can emerge from the noise in the partitioning of parent cell biomolecules among the daughter cells during cancer cell division. Our model captures the population-level behavior of murine prostate cancer cells, the hysteresis in the population-level dynamics of epithelial-mesenchymal plasticity, and how hybrid phenotype-promoting factors alter the phenotypic composition of a population. We further used the model to describe the implications of heterogeneity and plasticity for therapeutic regimens. By linking the dynamics of an intracellular regulatory circuit to the phenotypic composition of a population, the study contributes towards understanding how non-genetic heterogeneity can be generated and propagated from a small, homogeneous population, and towards therapeutic targeting of cancer cell heterogeneity and plasticity.

## Introduction

A tumor can contain cancer cells exhibiting multiple, distinct phenotypes. Subpopulations of cells exhibiting a stem cell-like phenotype have been reported in multiple cancer types including leukemia [1], breast cancer [2], colorectal cancer [3,4], brain cancer [5], and prostate cancer [6]. Tumor cell populations in triple-negative breast cancer can consist of luminal, basal, immunomodulatory, mesenchymal, and stem-like cells [7], while those in small cell lung cancer are composed of neuroendocrine and non-neuroendocrine cells [8]. Intra-tumoral phenotypic heterogeneity can have both genetic and non-genetic bases [9] and has been shown to be associated with poor prognosis across cancer types [10]. A non-genetic mechanism is likely to be the underlying cause if cancer cells within a tumor exhibit different phenotypes in spite of carrying the same genetic alterations. Further, cancer cells have been shown to be able to switch between different phenotypic states, either spontaneously or in response to specific cues. Spontaneous switching between luminal, basal, and stem-like states has been reported in breast cancer cell lines [11] while androgen-deprivation therapy has been shown to promote transition to a neuroendocrine phenotype in prostate cancer [12,13]. Cancer cells with different phenotypes can exhibit different sensitivities to various drugs [14,15]. Therefore, non-genetic mechanisms of phenotypic heterogeneity and plasticity in cancer cells are a fundamental challenge to anti-cancer therapies and an understanding of such mechanisms is essential to the development of effective anti-cancer treatments.

A canonical example of non-genetic heterogeneity is the phenotypic heterogeneity arising due to epithelial-mesenchymal plasticity (EMP). This plasticity involves two reversible processes, epithelial-mesenchymal transition (EMT) and mesenchymal-epithelial transition (MET). Via EMT, cancer cells, to varying extents, can lose epithelial characteristics such as cell-cell adhesion and apico-basal polarity, and acquire mesenchymal features which allow cancer cells to migrate effectively and invade. The reverse change is observed during MET—cells lose their migratory freedom and re-acquire epithelial hallmarks including expression of junctional proteins [16]. Recent studies have shown that cancer cells can also stably exist in a hybrid epithelial-mesenchymal state wherein they co-express epithelial and mesenchymal markers [17–19]. Cancer cells within a solid tumor exhibit widespread heterogeneity in the expression of epithelial and mesenchymal markers and can acquire an epithelial (E), a mesenchymal (M), or one or more hybrid epithelial-mesenchymal (hybrid E / M) phenotype(s) [20–23].

By allowing the tumor cells to acquire migratory traits for dissemination to distant organs, EMP plays a key role in the metastatic spread of solid tumors. The disseminated cells can then re-acquire epithelial traits including cell-cell adhesion to establish a tumoral mass at the new organ site. EMP has further been implicated in the triggering of stemness programs in cancer cells [24,25], evasion of the host immune response [26], and emergence of resistance to anti-cancer therapies [27–29]. Hybrid E / M cells play a key role in the collective dissemination of tumor cells as clusters, an aggressive mechanism of cancer metastasis [30]. Further, EMP-associated phenotypes differ in their tumor-seeding abilities [31,32] and in their sensitivity to drugs [33,34]. Understanding the mechanisms driving EMP will thus be a critical step in the development of more effective anti-cancer therapies.

Signaling pathways and the various transcription factors, micro-RNAs, and environmental stimuli driving EMP are well characterized [16]. The population-level dynamics of EMP, on the other hand, have only recently come into focus. Ruscetti *et al*. showed, in a mouse model of prostate cancer, that cells exhibiting a single EMP-associated phenotype can spontaneously generate a population with all three phenotypes over a period of time [35]. Celià -Terrassa *et al*. observed hysteresis in the dynamics of EMP in multiple normal and cancerous mammary epithelial cell lines and reported a role for hysteretic dynamics in the metastatic ability of cancer cells [36]. Risom *et al*. showed that drugs targeting specific cellular pathways can modulate transitions between epithelial-like and mesenchymal-like states in breast cancer cell lines [14]. The study thus established phenotypic plasticity as a therapeutically targetable property. The mechanism(s) responsible for cancer cells being able to switch between different phenotypic states in these studies, however, remain(s) uncharacterized. Some studies have described phenomenological, population-level models of phenotypic plasticity in cancer cells [11,14,37]. While predictions from these models fit the experimental data reported in the respective studies, such models lack detailed biomolecular mechanistic bases. This limitation makes the use of such phenomenological models difficult for making predictions directly useful in designing anti-cancer therapies.

Multiple mechanisms can drive phenotypic heterogeneity in a population. Apart from stochastic changes in the phenotypes of cells in a population due to intrinsic noise [38], heterogeneity can also arise from variable strengths of regulatory interactions in the cells in a population. The regulatory circuits in different cells can then exhibit distinct dynamics in response to the same external cues [39]. Another mechanism by which heterogeneity can emerge is cell-cell communication. A prominent example is Notch-Delta-Jagged signaling wherein cells in a population can exhibit a sender phenotype, a receiver phenotype, or a hybrid sender-receiver phenotype [40]. Here, we focus on the emergence of heterogeneity via the first mechanism, i.e., stochastic changes in the phenotypes of cells in response to noise.

Stochastic changes in cell phenotype, in general, require a mechanism to generate noise and a mechanism to stabilize the decision reached in response to the noise [41]. In both prokaryotes and eukaryotes, noise can arise from the inherent stochasticity of biochemical reactions. A typical case is the emergence of antibiotic-resistant persister cells in bacterial populations [42]. Another source of noise is the random partitioning of parent cell biomolecules among the daughter cells at the time of cell division [43]. A key role for such noise in the generation of non-genetic heterogeneity has been proposed [44]. Fluctuations in the cell phenotype in response to noise are usually small and transient. Therefore, stochastic changes in cell phenotype also require a mechanism that can amplify the fluctuations in the phenotype in response to the noise. One such mechanism is afforded by the multi-stability of the mechanism underlying cell fate determination. In such a scenario, even small fluctuations added to a stable phenotypic state can cause the cell to transition to a new stable phenotypic state. In comparison to their non-malignant counterparts, cancer cells divide uncontrollably, making random partitioning of biomolecules during cell division a significant source of noise. In the context of cancer cells, the regulatory circuit governing EMP has been shown to exhibit tri-stability [45]. Thus, both the requirements for stochastic changes in cell phenotype are satisfied for a population of cancer cells exhibiting EMP.

Here, we show that in a population of cells each carrying a copy of the EMP regulatory circuit, phenotypic heterogeneity can arise from the noise generated due to the random partitioning of micro-RNAs, mRNAs, and transcription factors at the time of cell division. The temporal dynamics of fractions of different phenotypes in a population predicted by our model agree with those observed for cells isolated from a mouse model of prostate cancer [35], both in terms of the timescale over which the phenotypic composition of the population changes and in terms of the phenotypic composition observed over time when starting from different initial conditions. Our model captures the relative stability of epithelial and mesenchymal populations and the ability of a hybrid E / M population to quickly generate a mixture of epithelial and mesenchymal cells. We also used the model to describe hysteresis in the population-level dynamics of EMP and characterized how EMP modulators such as GRHL2 and ΔNP63*α* can alter the phenotypic composition of a population. Our model predicted that a suitable combination of a MET promoter, such as retinoic acid, and an EMT promoter, such as TGF-*β*, can stabilize a population of hybrid E / M cells. Finally, we characterized how drugs targeting specific EMP-associated phenotypes alter the population behavior. We conclude that targeting a single phenotype is likely to have little attenuating influence on tumor size. Targeting epithelial and mesenchymal cells simultaneously is predicted to be the best regimen for restricting tumor growth.

## Results

### Developing a population-level model of EMP dynamics

**Fig 1:**
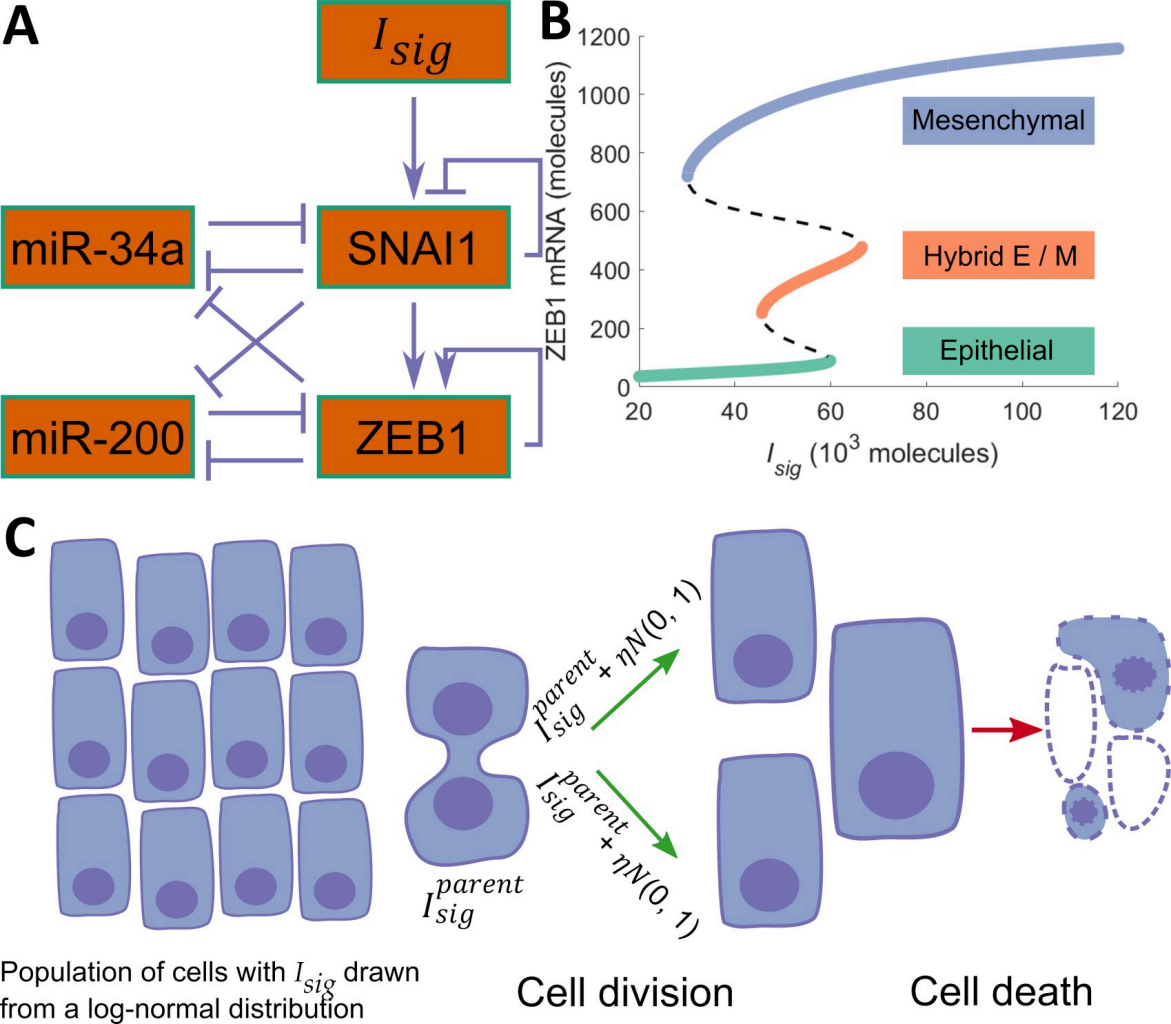
Description of the proposed model. (A) The regulatory circuit governing EMT and MET. (B) Bifurcation diagram for the EMP regulatory circuit. (C) A schematic of the simulations carried out in the present study. Each cell carries a copy of the regulatory circuit shown in Fig 1(A).

Epithelial-mesenchymal plasticity (EMP) is modulated via the functional cooperation of multiple cell signaling pathways that can respond to both internal and external stimuli [46]. These include TGF-*β*-SMAD3, WNT-*β*-catenin, and Notch pathways. The activities of these pathways converge onto a core regulatory circuit shown in Fig 1(A). Downstream targets of this circuit include cadherins, claudins, occludins, and metalloproteinases [46]. Given its central role in modulating EMP [16,46], we used this regulatory circuit to construct a model of epithelial-mesenchymal heterogeneity in cancer cells. The EMP regulatory circuit forms a ternary switch, the dynamics of which has been described previously [45]. Briefly, the circuit can exhibit three distinct types of stable steady states in response to different levels of the *SNAI1* activating signal *I*_*sig*_. The stable steady state types can be mapped to the three EMP-associated phenotypes: epithelial (E), mesenchymal (M), and hybrid epithelial-mesenchymal (hybrid E / M) (Fig 1(B)). We considered a population of cancer cells where each cell is carrying a copy of this EMP regulatory circuit. The kinetic parameters governing the behavior of the regulatory circuit have been described previously [45]. The parameters are such that the circuit operates in the tri-stable region. This choice of parameters allowed us to explore the behavior of all three EMP-associated phenotypes and is suited for describing experiments characterizing epithelial-mesenchymal heterogeneity. The dynamics of the EMP regulatory circuit within each cell can be simulated independent of the other cells in the population. Each cell can then be assigned one of the three phenotypes based on the expression level of ZEB1 mRNA inside the cell. In the absence of competition (or, equivalently, in the presence of an infinite supply of nutrients), each cell in the population had an average doubling time of *38* hours which is a typical value for cancer cells (BioNumbers ID 100685 [47]). To account for the limited availability of nutrients in the tumor microenvironment, we used a logistic growth model with a fixed carrying capacity. Cells in the population died at a fixed rate. Different EMP-associated phenotypes can exhibit different rates of cell division. Induction of EMT has been shown to arrest the cell cycle [48,49]. Another study has shown that cells that have undergone a partial EMT can exhibit a hyper-proliferative phenotype [50]. Here, we considered a simpler case wherein both division and death rates are assumed to be independent of the phenotype of the cell. Relaxing this assumption does not affect the general conclusions presented in this study (S9–S12 Figs). When a cell divides, the RNA and protein molecules present in the parent cell right before cell division are randomly partitioned among the daughter cells. *I*_*sig*_ represents multiple upstream signaling pathways whose functionalities converge onto the EMP regulatory circuit and is the chief determinant of the number and types of stable steady states the EMP regulatory circuit can acquire. Therefore, noise in the partitioning of *I*_*sig*_ among the daughter cells is likely to be the dominant perturbation to the dynamics of the EMP regulatory circuit and is the focus of our proposed model. The concentration of *I*_*sig*_ in each daughter cell after cell division was given by:

$$I_{sig}^{daughter}=I_{sig}^{parent}+\eta N\left(0,1\right)$$ (1)

Here, *N*(0,1) is the standard normal distribution and *η* is a model parameter controlling the variance of the noise. See S1 Text (section I) for a detailed description of development of this model of partitioning noise. Briefly, right before a cell divides, its genomic content is duplicated and, consequently, the copy numbers of different RNA and protein molecules get approximately doubled. During cell division, these molecular species are randomly partitioned among the daughter cells. Thus, there are two independent sources of noise involved in each cell division event—noise in RNA and protein copy number doubling in the parent cell during the duplication of its genomic content and noise due to the random partitioning of RNA and protein molecules among the daughter cells during cell division. To incorporate both these noise sources, our model, represented by Eq 1, uses two independent random variables (one random variable in the equation for each daughter cell) to describe each cell division event. As mentioned earlier, *I*_*sig*_ is representative of multiple signaling pathways and each such pathway involves numerous molecular players. The normally distributed noise term in Eq 1 accounts for the contributions from noise in the partitioning of these molecular players. Such a coarse grained approach, instead of more exact formulations described previously [43,51], becomes necessary due to the large number of molecular players involved in EMT / MET signaling, mechanisms of action of many of which remain uncharacterized.

With the new concentration of *I*_*sig*_, as determined by Eq 1, the EMP regulatory circuit in the daughter cell will acquire a stable steady state different from the stable steady state of the EMP regulatory circuit in the parent cell. The new stable steady state may correspond to a phenotypic state different from the phenotypic state of the parent cell (Fig 1(B)). The daughter cell can then acquire a phenotype different from the phenotype of the parent cell before division. A schematic of the model is shown in Fig 1(C).

### Changes in phenotype during cell division are governed by the Noise in *I*_*sig*_ partitioning

**Fig 2:**
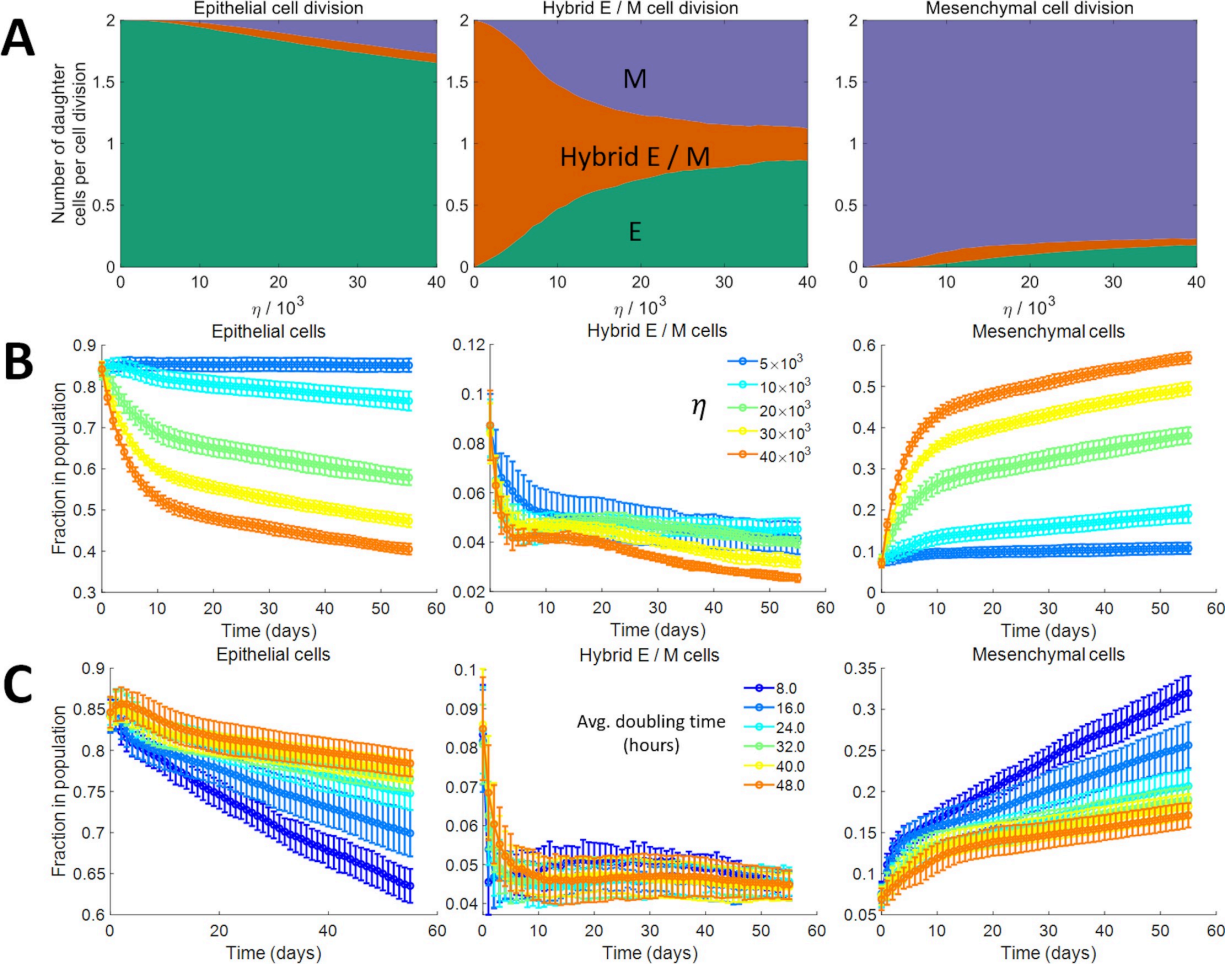
Dynamics of the model. (A) Average number of daughter cells of each phenotype generated per cell division when the parent cell was an epithelial cell (left panel), a hybrid E / M cell (center panel), or a mesenchymal cell (right panel). (B) Temporal dynamics of the fraction of epithelial cells, hybrid E / M cells, and mesenchymal cells in a population of cancer cells for different values of the noise parameter *η* (indicated by different colors). Here, the average doubling time of each cell was *38*.*0* hours. (C) Temporal dynamics of the fraction of epithelial cells, hybrid E / M cells, and mesenchymal cells in a population of cancer cells for different average doubling times of cells in the population (indicated by different colors). Here, *η* = *1*.*0*×*10*^*4*^. In each panel of (B) and (C), the results shown were obtained by averaging over *16* distinct simulation runs. Error bars indicate the standard deviation calculated over the *16* independent runs.

We started with a population of *500* cells on day *0*. The initial concentration of *I*_*sig*_ in these cells was drawn from a log-normal distribution [52] with median *2×10^4^* molecules / cell and with coefficient of variation *1*.*0*. The dynamics of this population was simulated for a period of *8* weeks using the model of EMP described above, for different values of the noise parameter *η*. The cells grew quickly in number, with the total number of cells in the population becoming nearly stationary around day *18*. The growth kinetics, as expected, were independent of the noise parameter *η*, and depended on the doubling time of cells in the population (S1 Fig). Every time a cell divided, we recorded the phenotypes of the parent and the daughter cells. At low values of *η*, cells of all three phenotypes exhibited high rates of symmetric self-renewal, i.e., both the daughter cells acquired the same phenotype as the parent cell in most instances (Fig 2(A)). As *η* was increased, the probability of a daughter cell acquiring a phenotype different from that of the parent cell increased for all three types of cells. For *η = 1*×*10*^*4*^, there was a ~*3%* chance that a dividing epithelial cell will generate at least *1* daughter that is non-epithelial. The chances increased to ~*17%* for *η* = *4*×*10*^*4*^. Similarly, for both hybrid E / M and mesenchymal cells, the probability of generating at least one daughter cell with a phenotype different from that of the parent cell increased significantly with an increase in *η*. Among the three phenotypes, hybrid E / M cells exhibited the highest probability of generating a daughter cell with a phenotype different from that of the parent cell. Even at low values of *η*, the probability that at least one of the daughter cells will be non-hybrid E / M when a hybrid E / M cell divided was as high as *50%*. This probability increased further with an increase in *η*, reaching ~*87%* for *η* = *4*×*10*^*4*^. On the other hand, mesenchymal cells exhibited the least probability of generating a daughter cell with a phenotype different from that of the parent cell. The probability of generating a non-mesenchymal cell during mesenchymal cell division did not exceed *12%* for any value of the noise parameter *η* for which model behavior was analyzed. The high probability of a daughter cell acquiring a phenotype different from that of the parent cell during hybrid E / M cell division is a consequence of the small range of *I*_*sig*_ concentrations for which the hybrid E / M phenotype can exist as a stable state (Fig 1(B)). In contrast, ranges of *I*_*sig*_ concentrations for which epithelial and mesenchymal phenotypes can exist as stable states are much larger. As a result, in most cell divisions involving an epithelial or a mesenchymal cell, daughter cells acquire the phenotype of the parent cell.

The model can predict how the fractions of different phenotypes in a population of cells will evolve over time, given the initial phenotypic composition of the population. Examples of temporal dynamics for different values of *η* are shown in Fig 2(B). On starting with a population that is ~*84%* epithelial, the fraction of epithelial cells in the population declined over time. The rate of this decline increased with increasing *η* due to the increase in the probability of generating a non-epithelial daughter cell during epithelial cell division. The fraction of mesenchymal cells, on the other hand, increased with time as mesenchymal cells were generated via the division of epithelial cells. Given the high probability of symmetric self-renewal of mesenchymal cells even at high values of *η*, the fraction of these cells in the population increased with increasing *η*. The temporal dynamics of the hybrid E / M fraction was non-monotonic and, due to the high probability of generating a non-hybrid E / M cell during the division of a hybrid E / M cell, their fraction in the population remained below *10%*.

In addition to the noise parameter *η*, average doubling time of cells in the population is also a key determinant of kinetics of change in the phenotypic composition of the population in our model (Fig 2(C)). Phenotypic composition of the population changed at a faster rate when the average doubling time of cells in the population was decreased (i.e., when the growth rate of cells in the population was increased). This is because the phenotypic composition of the population in our model changes due to the possibility of a daughter cell acquiring a phenotypic state different from that of the parent cell. As a result, change in the phenotypic composition of the population is accelerated on increasing the rate of cell division. Increasing the death rate of cells in the population also increased the rate of change in the phenotypic composition of the population (S2 Fig). This behavior is due to the increased frequency of cell division events in the steady state population under higher death rates. The effect of change in death rate of cells was, however, less pronounced than the effect of change in the growth rate of cells.

Our results suggest that the phenotypic composition of an isogenic population of cells *in vitro* at a given point in time will depend on the phenotypic composition at the time point the population was initially sorted or passaged and on the number of days since the sorting or passaging event (Fig 2(B) and 2(C); S3(A) Fig). Many cell lines commonly used in experimental studies to investigate EMP in cancer cells are known to exhibit epithelial-mesenchymal heterogeneity and cells are often sorted in studies to isolate those exhibiting a desired phenotype. Our model suggests that the phenotypic heterogeneity exhibited by a given cell line can vary from study to study even under control conditions (cells untreated with any reagent that may promote or inhibit EMT / MET) and be propagated post sorting of cells. To investigate if such behavior is observed, we pooled publicly available gene expression data for nine cell lines and used these expression data to calculate EMT scores for each cell line as described previously [53]. The EMT score indicates how epithelial-like or mesenchymal-like a cell line is. S3(B) Fig shows that the EMT score calculated for a given cell line under untreated conditions can indeed vary significantly across independent experimental studies. Multiple factors, including cell passaging technique, gene expression profiling method, and technical variability may be held responsible for the existence of these variations. Our model provides another possible, rather straightforward, explanation of such behavior—in different experimental studies involving a given cell line, phenotypic composition post cell passage / sorting is likely to be different and gene expression profiling was likely carried out at different time points post passaging / sorting of cells.

Finally, spontaneous change in the phenotypic composition of a population over time can occur as long as the regulation of EMP exhibits multi-stability in response to an upstream signal. Our use, in this study, of the model of EMP regulation described by Lu *et al*. [45] (Fig 1(A) and 1(B)) is only a convenient choice. We also used our model of partitioning noise (represented by Eq 1) to simulate the dynamics of populations when incorporating two distinct models of EMP regulation—a simplistic two-state model with no hybrid E / M phenotype [36] (S4(A) Fig) and a four-state model with OVOL2-ZEB1 mutual inhibition and two hybrid E / M phenotypic states [54] (S5(A) Fig). In both scenarios, the phenotypic composition of the population changed spontaneously over time upon the inclusion of noise in the partitioning of the EMT activator during cell division (S4(B) Fig and S5(B) Fig).

### Model captures the temporal changes in phenotypic composition in a mouse model of prostate cancer

**Fig 3:**
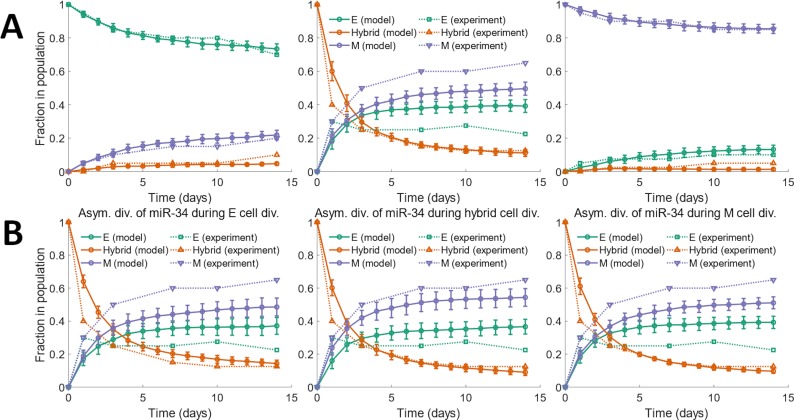
Comparison of temporal dynamics of phenotypic composition of a population predicted by our model with experimental data obtained for murine prostate cancer cells. (A) Fractions of different phenotypes in the population assessed at different time points during a two-week period. Dotted curves represent the fractions of different phenotypes re-plotted from Ruscetti *et al*. [35]. Solid curves, with circles, represent the predictions from our model using *η* = *2*.*7*×*10*^*4*^. Best fit to experimental data was obtained for this value of the noise parameter. (B) Phenotypic composition, over time, of a population which consisted of only hybrid E / M cells on day *0*. The three panels show the behavior under different modifications to the model—asymmetric distribution of miR-34a among the daughter cells during the division of epithelial cells (left panel; *η* = *2*.*3*×*10*^*4*^), asymmetric distribution of miR-34a among the daughter cells during the division of hybrid E / M cells (center panel; *η* = *2*.*5*×*10*^*4*^), and asymmetric distribution of miR-34a among the daughter cells during the division of mesenchymal cells (right panel; *η* = *2*.*7*×*10*^*4*^). The value of *η* indicated for each case resulted in the best fit to experimental data in that case. The average doubling time of cells in each panel was *38*.*0* hours. The results shown in each panel were obtained by averaging over *16* distinct simulation runs. Error bars indicate the standard deviation calculated over the *16* independent runs.

We tested the predictions of our model in tumor cells isolated from murine prostate tumors [35]. To study the role of EMP in the distant metastasis of prostate cancer *in vivo*, Ruscetti *et al*. crossed *Pb-Cre*^*+/-*^*; Pten*^*L/L*^*; Kras*^*G12D/+*^ mice with *Vim-GFP* reporter mice to track EMP in prostate cancer cells. EpCAM^+^GFP^-^ epithelial tumor cells were FACS (fluorescence-activated cell sorting) sorted from *10*-week old prostates and cultured *in vitro* (*PKV* cell line). FACS sorting of these cells after *14* days of *in vitro* culture revealed three distinct phenotypes: EpCAM^+^GFP^-^ (epithelial), EpCAM^-^GFP^+^ (mesenchymal), and EpCAM^+^GFP^+^ (hybrid E / M). Epithelial, mesenchymal, and hybrid E / M cells, FACS sorted from the *PKV* cell line, were then cultured *in vitro*. Fractions of different phenotypes in each culture at different time points were tracked using FACS. The temporal dynamics have been replotted in Fig 3 along with the predictions from our model.

Model predictions shown in Fig 3(A) were obtained by varying the noise parameter *η* to minimize the root mean square deviation from experimental data (S6(B) Fig). Best fit to experimental data was obtained for *η* = *2*.*7*×*10*^*3*^. The ratio of the noise in the partitioning of *I*_*sig*_ (*η*) to the mean *I*_*sig*_ of dividing cells in this case was estimated from the simulations to be ~*0*.*38*. When multiple activating and inhibitory inputs to the core EMP regulatory circuit are considered individually, best fit to experimental data may be obtained at a much lower value of the noise parameter *η* (S7 Fig). Our model was able to capture the time scale over which the phenotypic composition of the population changed in each of the three cases with different initial compositions. When starting with a population consisting of only epithelial cells, ~*73%* of cells were epithelial at the end of the *14*-day simulation and experiment period. Consistent with the high rate of symmetric self-renewal of mesenchymal cells, nearly *85%* of the population was still mesenchymal after the *14*-day period when starting with a population of only mesenchymal cells on day *0*. On the other hand, on starting with a population of hybrid E / M cells on day *0*, the fraction of these cells in the population dropped below *30%* within *3* days. A mixture of epithelial and mesenchymal cells had quickly been generated by the population of hybrid E / M cells.

One aspect of the experimental behavior shown by the *PKV* cell line is not captured by our baseline modeling framework. Hybrid E / M cells isolated from the *PKV* cell line, when cultured *in vitro*, generated a population with more mesenchymal cells than epithelial cells (Fig 3(A); center panel, dotted curves). This behavior was not captured by our model which treats epithelial, mesenchymal, and hybrid E / M cells equivalently, i.e., doubling times, death rates, and the characteristics of noise in the partitioning of *I*_*sig*_ during cell division are identical for cells of all three phenotypes in our model. Hybrid E / M cells, however, exhibit a special property—multiple studies have reported that the hybrid E / M phenotype is associated with the expression of cancer stem cell markers [24,55–57]. During the division of colon cancer stem cells [58–60] and of mammary stem cells [61], asymmetric partitioning of miR-34a among the daughter cells has been reported. Bu *et al*. [58] have shown that miR-34a is asymmetrically divided among the daughter cells during the asymmetric division of colon cancer stem cells (see Fig 3(A) and 3(B) in Bu *et al*. [58]). This was later confirmed in a couple of follow-up studies (see Fig 1(A) in Bu *et al*. [59] and Fig 4(E) in Wang *et al*. [60]). Hybrid E / M cells in our modeling framework exhibit an intermediate level of miR-34a expression while epithelial cells have high miR-34a expression and mesenchymal cells have low miR-34a expression (S6(A) Fig). Bu *et al*. [58] showed that an intermediate level of miR-34a expression is needed for the asymmetric division of colon cancer stem cells during which miR-34a is asymmetrically partitioned among the daughter cells. Both ectopic expression of miR-34a and knock down of miR-34a inhibit asymmetric division (see Fig 2(E) and 2(F) in Bu *et al*. [58]).

On the basis of the experimental observations reported above, we incorporated the stemness property of hybrid E / M cells into our model by setting the concentration of miR-34a in one of the daughter cells to zero during the division of a hybrid E / M cell while keeping the concentration of miR-34a in the other daughter cell same as the miR-34a concentration in the parent cell. Here, the assumption is that miR-34a is actively and quickly degraded in one of the daughter cells (the one for which miR-34a is set to zero) right after division. When simulating the dynamics of a population with only hybrid E / M cells on day *0*, this modification to the model led to a larger fraction of mesenchymal cells as compared to epithelial cells in the population at subsequent time points. This modification allowed for a better fit to the experimental observations obtained for cells isolated from the *PKV* cell line (Fig 3(B); center panel; S6(B) Fig). Model behavior is unchanged if during the division of a hybrid E / M cell, one of the daughter cells gets no miR-34a molecules while the other daughter cell gets double the concentration of miR-34a in the parent cell (due to the doubling of miR-34a copy number in the parent cell right before it divides; S14 Fig). Since epithelial cells have high miR-34a expression and mesenchymal cells have low miR-34a expression (S6(A) Fig), both these cell types are unlikely to exhibit asymmetric partitioning of miR-34a during cell division [58]. In any case, asymmetric partitioning of miR-34a among the daughter cells during the division of epithelial or mesenchymal cells did not typically lead to a better fit to experimental data (Fig 3(B); left and right panels; S6(B) Fig).

**Fig 4:**
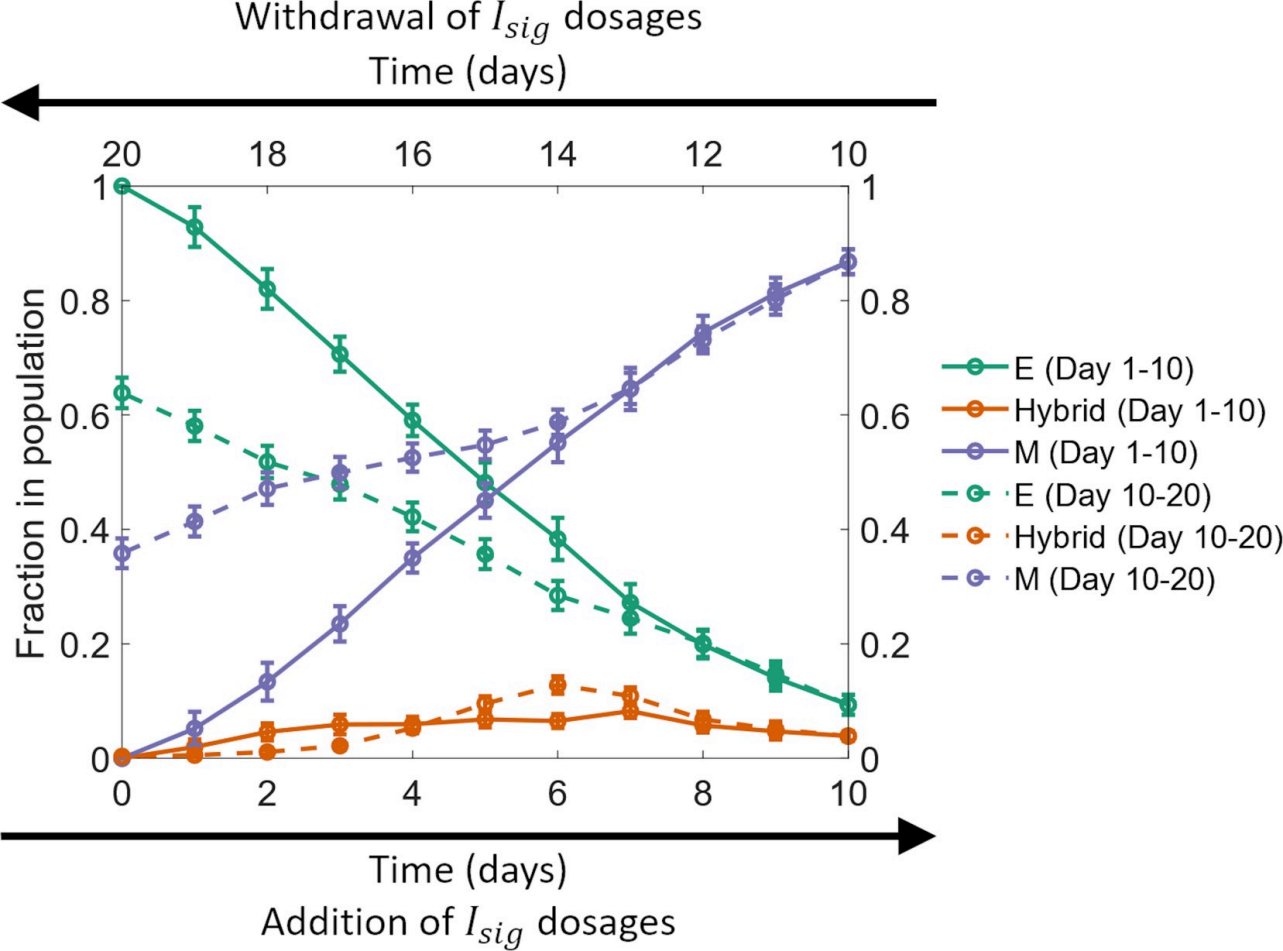
Hysteresis in the temporal behavior of fractions of different phenotypes. Solid curves show the behavior from day *0* to day *10* (bottom horizontal axis; left to right) and the dashed curves show the behavior from day *11* to day *20* (top horizontal axis; right to left). Here, *η* = *2*.*7*×*10*^*4*^ and the average doubling time of cells was *38*.*0* hours. The results shown here were obtained by averaging over *16* distinct simulation runs. Error bars indicate the standard deviation calculated over the *16* independent runs.

Our baseline modeling framework, built upon the canonical EMT / MET regulatory network, describes generic epithelial-mesenchymal plasticity (EMP) behavior. The above paragraph illustrates how the modeling framework can be modified to incorporate any special property (asymmetric partitioning of miR-34a for example) that cancer cells in a given phenotypic state may exhibit. Thus, our framework can easily be adapted to fit experimental data in diverse contexts where cancer cells may exhibit peculiar characteristics that modify their generic EMP behavior.

Note that since the kinetics of change in the phenotypic composition of a population also depend on the average doubling time of cells in the population (Fig 2(C)), one may vary this rate while keeping the noise parameter *η* fixed to obtain the best fit to the experimental data from Ruscetti *et al*. [35]. Model predictions for the average doubling time of cells that minimizes the root mean square deviation from experimental data are shown in S8 Fig.

We also investigated how the model behavior will change if we take into account the different growth phenotypes that may be exhibited by hybrid E / M cells—a hyper proliferative phenotype [50] (S9(A) Fig) or a growth-arrested phenotype [48,49] (S9(B) Fig). In both scenarios, a population of hybrid E / M cells exhibits plastic behavior, quickly generating a mixture of epithelial and mesenchymal cells. The decay in the fraction of hybrid E / M cells in the population is accelerated when hybrid E / M cells are hyper proliferative (average doubling time of *8*.*0* hours) and slowed when hybrid E / M cells are growth arrested (average doubling time of *192*.*0* hours or *8* days). Behavior of populations that were purely epithelial or purely mesenchymal on day *0* is unchanged in both scenarios. Effects of co-varying the average doubling times of cells exhibiting different phenotypes are shown in S10–S12 Figs. Finally, temporal behavior of the model was unaffected when noise in the partitioning of other players in the core EMP regulatory circuit was also incorporated into the simulations (S13 Fig).

### Hysteresis in the population-level dynamics of epithelial-mesenchymal plasticity

The regulatory circuit driving epithelial-mesenchymal plasticity, shown in Fig 1(A), exhibits nonlinear dynamics. Such systems can exhibit hysteresis wherein the system response depends not only on the present input but also on the input history. We investigated if hysteresis can also arise in the dynamics of fractions of different phenotypes in a population of cancer cells. Starting with a population of epithelial cells on day *0*, a fixed dosage of *I*_*sig*_ was added each day for *10* days. A fixed dosage of *I*_*sig*_ was then withdrawn each day over the next *10* days. Fig 4 shows the fractions of different phenotypes in the population over the *20* day period. Since *I*_*sig*_ promotes EMT, the fraction of mesenchymal cells in the population increased during the first *10* days, from *0%* on day *0* to >*86%* on day *10*. In contrast, the fraction of epithelial cells declined to less than *10%* over the same time period. When *I*_*sig*_ was withdrawn in fixed dosages day *11* onwards, this trend was reversed. However, the fractions of epithelial and mesenchymal cells in the population from day *11* to day *20* did not retrace the behavior recorded from day *0* to day *10*. On day *20*, >*35%* of the cells still exhibited a mesenchymal phenotype which was maintained in the absence of additional *I*_*sig*_ dosages (S15 Fig). These cells thus represented a population that had undergone an EMT which is irreversible on the time scale considered here. Such hysteretic control of EMT and MET dynamics was recently confirmed in multiple normal and cancerous mammary epithelial cell lines including MCF10A, MCF7, HMLE, T47D, and 4T1 [36]. The study also confirmed the persistence of the mesenchymal phenotype long after the withdrawal of the EMT-inducing signal as predicted by our model (S15 Fig).

### Frequency of spontaneous phenotype change during cell division can be modulated by altering the core EMP regulatory circuit

**Fig 5:**
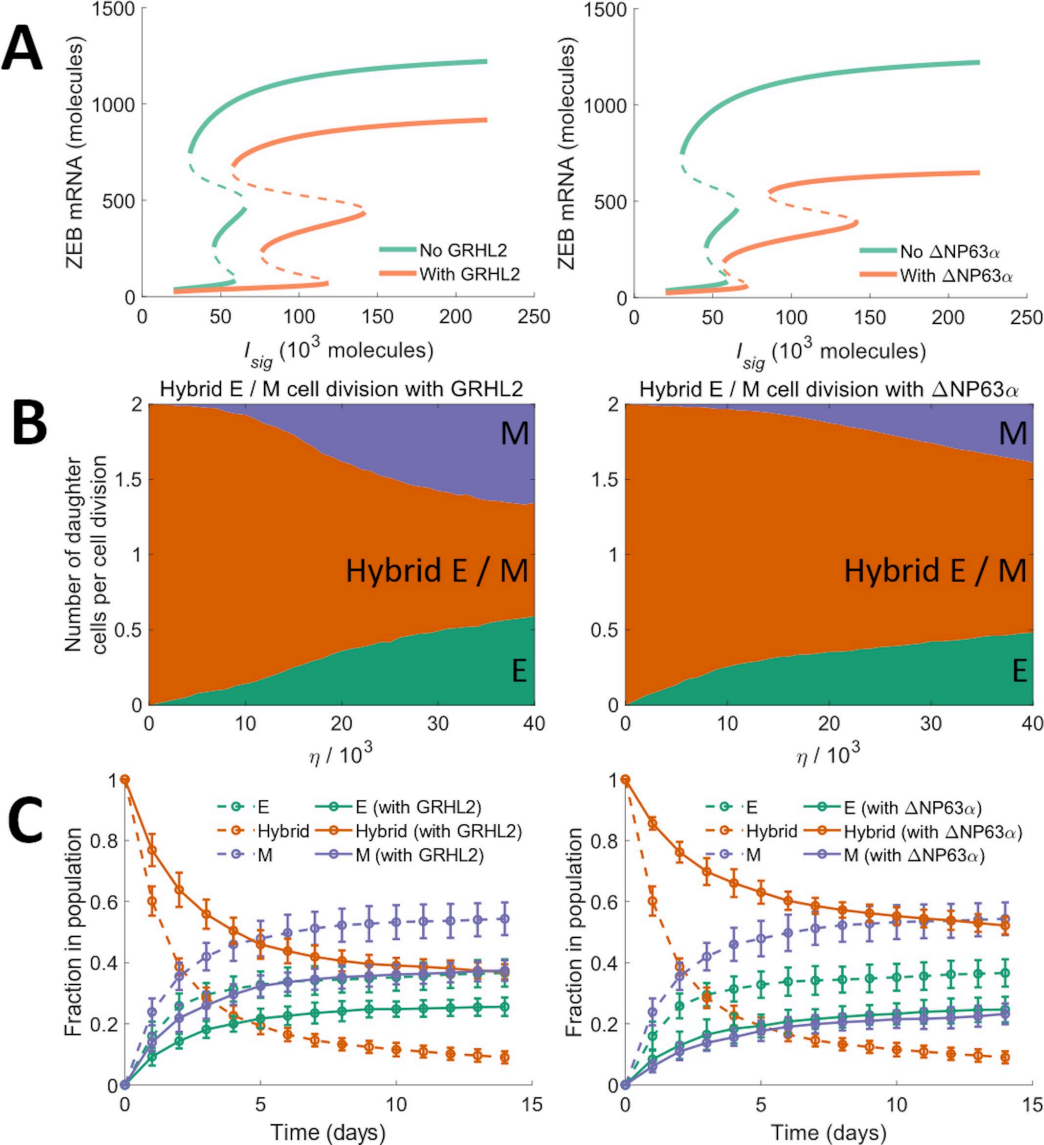
Model dynamics in the presence of EMT modulators. (A) Bifurcation diagrams of the core EMP regulatory circuit in the presence of GRHL2 (left panel) and in the presence of ΔNP63*α* (right panel). (B) Average number of daughter cells of each phenotype generated per cell division during the division of a hybrid E / M cell in the presence of GRHL2 (left panel) and in the presence of ΔNP63*α* (right panel). (C) Fractions of different phenotypes, over time, in a population which consisted of only hybrid E / M cells on day *0*. Solid curves in the left panel show the behavior in the presence of GRHL2 and solid curves in the right panel show the behavior in the presence of ΔNP63*α*. Dotted curves in left and right panels indicate the fractions of different phenotypes in the absence of GRHL2 and ΔNP63*α* activity respectively. Here, *η* = *2*.*7*×*10*^*4*^ and the average doubling time of cells was *38*.*0* hours. The results shown in both panels were obtained by averaging over *16* distinct simulation runs. Error bars indicate the standard deviation calculated over the *16* independent runs.

In our model, changes in phenotype during cell division occur, in response to noise, due to the tri-stability in the dynamics of the core EMP regulatory circuit. Thus, modifications to this regulatory circuit which change the bifurcation diagram (Fig 1(B)) should alter the frequency of phenotype change during cell division. Mathematical models have shown that the range of *I*_*sig*_ levels for which a stable hybrid E / M phenotype can exist is much larger when GRHL2 is coupled with the core EMP regulatory circuit [17] or when ΔNP63*α* is added to the regulatory circuit [62] (Fig 5(A)). Given the larger range of *I*_*sig*_ levels for which a stable hybrid E / M phenotype can exist in both these scenarios, we expected the probability of generating an epithelial or mesenchymal daughter cell during the division of a hybrid E / M cell to decrease upon the inclusion of GRHL2 or ΔNP63*α* in our model. This was confirmed by tracking the phenotypes of daughter cells formed via the division of a hybrid E / M cell upon the inclusion of GRHL2 or ΔNP63*α*. For all values of the noise parameter *η* investigated, a higher probability of generating at least *1* hybrid E / M daughter cell during the division of a hybrid E / M cell was observed upon the inclusion of both GRHL2 and ΔNP63*α* (Fig 5(B)). Even at high values of *η* (*η* = 4×10^4^), inclusion of ΔNP63*α* did not let the probability of generating at least *1* non-hybrid E / M daughter cell exceed *44%*. This probability was >*86%* in the absence of ΔNP63*α*. Upon starting with a population of hybrid E / M cells on day *0*, nearly *37%* of cells exhibited a hybrid E / M phenotype on day *14* upon the inclusion of GRHL2 as compared to less than *12%* of cells in the absence of GRHL2 activity (Fig 5(C); left panel). When ΔNP63*α* was included, the fraction of hybrid E / M cells in the population on day *14* was >*50%* (Fig 5(C); right panel), a fraction larger than the one seen in the presence of GRHL2 activity and larger than the fraction seen in the absence of both GRHL2 and ΔNP63*α*. This behavior is reminiscent of the increased mean residence time of cells in a hybrid E / M state in the presence of GRHL2 and ΔNP63*α* which has been reported previously [63].

**Fig 6:**
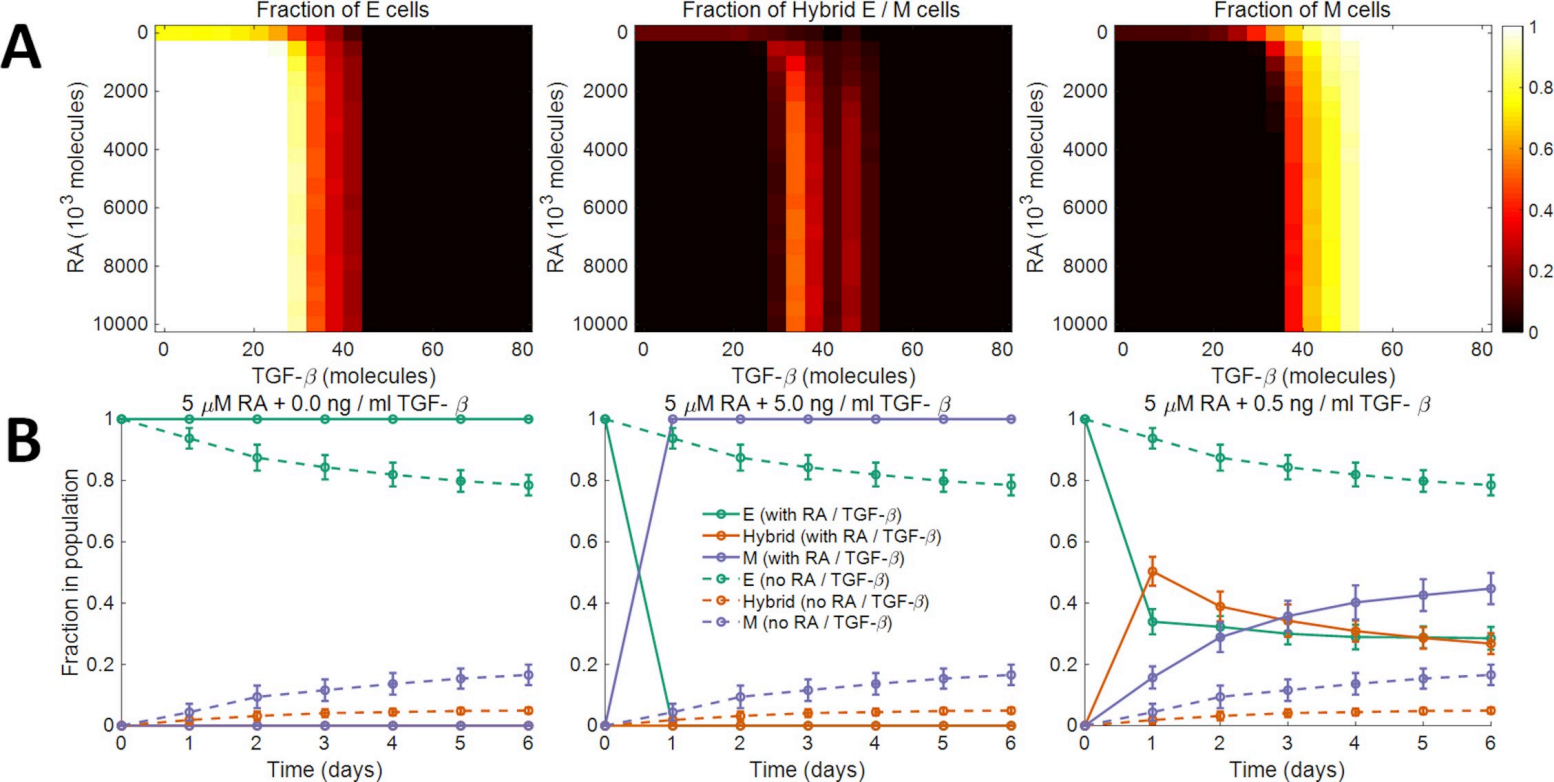
Model dynamics in the presence of retinoic acid and TGF-*β*. (A) Fractions of epithelial (left panel), hybrid E / M (center panel), and mesenchymal (right panel) cells in the population after *14* days upon co-treatment with different concentrations of retinoic acid (RA) and TGF-*β*. The population consisted of only epithelial cells on day *0*. (B) Biddle *et al*. [64] characterized the plasticity of oral squamous cell carcinoma cells in response to co-treatment with different concentrations of retinoic acid (RA) and TGF-*β*. *5*.*0* μM RA blocked EMT. Its effect was completely abrogated by the addition of *5*.*0* ng / ml of TGF-*β*. Co-treatment with *5* μM RA and *0*.*5* ng / ml TGF-*β* established a regime in which a population of cells that have undergone a partial EMT was maintained. Starting with a population of epithelial cells on day *0*, we simulated the dynamics under these three co-treatment regimens (solid curves in the three panels). The predictions from our model are in agreement with the experimental observations of Biddle *et al*. Dashed curves indicate the dynamics in the absence of both RA and TGF-*β*. Here, *η* = *2*.*7*×*10*^*4*^ and the average doubling time of cells was *38*.*0* hours. The results shown in each panel were obtained by averaging over *16* distinct simulation runs. Error bars indicate the standard deviation calculated over the *16* independent runs.

We next investigated how other modulators of EMT or MET may alter the phenotypic composition of a population of cells. Biddle *et al*. used co-treatment with *5 μ*M retinoic acid and *0*.*5* ng / ml TGF-*β* to maintain a subpopulation of cells in the partial EMT state for two oral squamous cell carcinoma cell lines [64]. We used our model to characterize the effects of co-treatment with retinoic acid, an MET inducer [65], and exogenous TGF-*β*, an EMT inducer [16], on the phenotypic composition of a population of epithelial cells. Starting with a population of epithelial cells on day *0*, we simulated the model for different concentrations of retinoic acid and exogenous TGF-*β*. Fig 6(A) shows the fractions of epithelial, mesenchymal, and hybrid E / M cells in the population on day *14*. The model predicts that using specific combinations of retinoic acid and TGF-*β* concentrations, a population with a large fraction of cells that have undergone a partial EMT can be maintained, thereby recapitulating the experimental observations of Biddle *et al*. [64] (Fig 6(B)).

### Drugs targeting epithelial and mesenchymal cells have a synergistic effect in restricting tumor size

**Fig 7:**
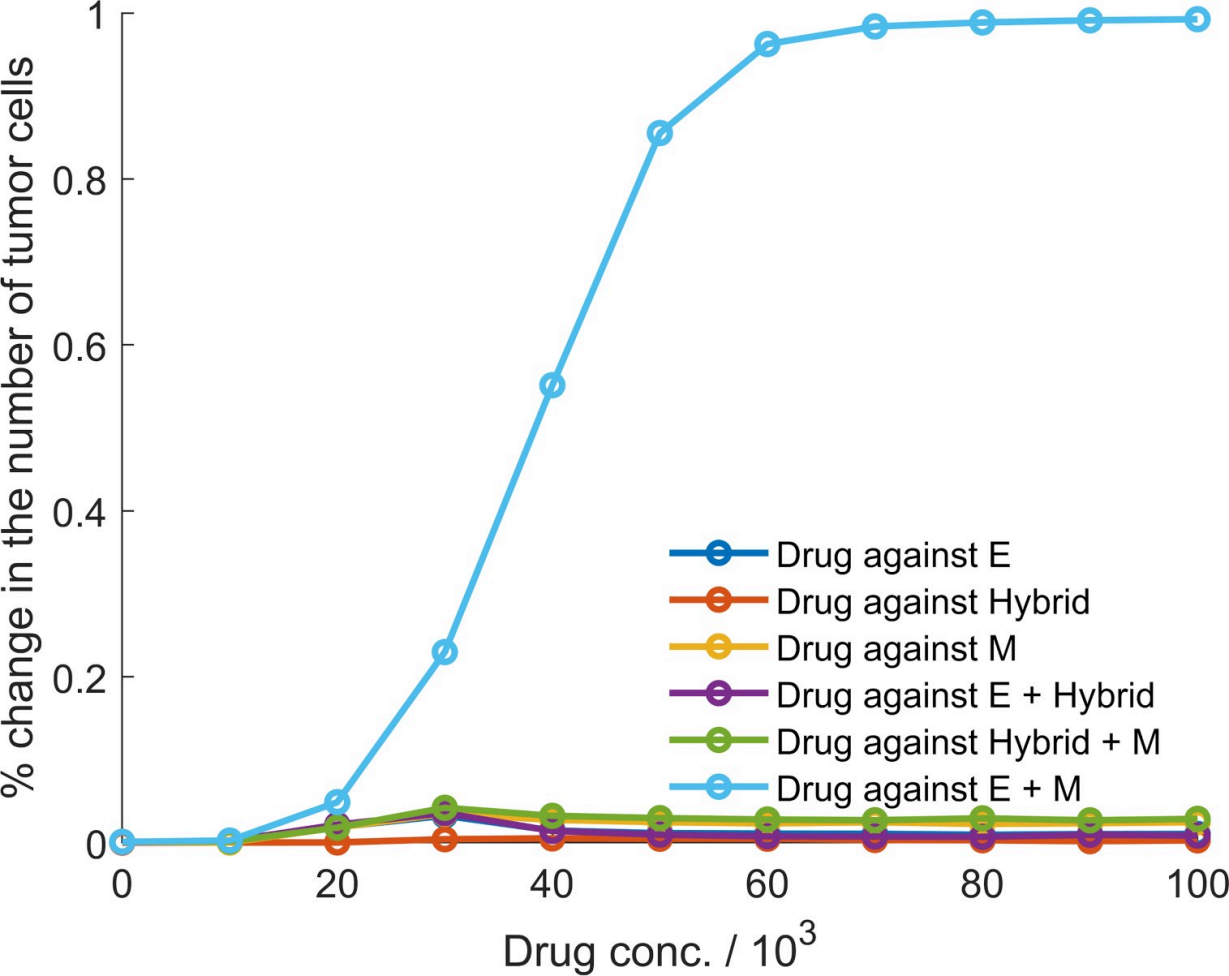
Percentage change in the number of tumor cells after drug treatment for *28* days. Different colors indicate different treatment regimens.

Finally, we investigated the effectiveness of targeting different phenotypes or phenotype combinations in restricting tumor growth. Drug activity was modeled using the shifted Hill function which altered the death rate of cancer cells by a multiplicative factor. Fig 7 shows how the number of tumor cells in the population changed under different drug regimens. Drugs targeting a single phenotype, whether epithelial, mesenchymal, or hybrid E / M, had a negligible effect on tumor size, even at high concentrations. Since epithelial cells can generate a population of mesenchymal cells, targeting the epithelial phenotype led to an increase in the fraction of mesenchymal cells in the population. These cells exhibit a low probability of generating non-mesenchymal daughter cells (Fig 2(A); right panel) and had greater nutrient availability once the population of epithelial cells had been depleted by the drug. Similarly, targeting of mesenchymal cells increased the fraction of epithelial cells in the population while doing little to restrict the tumor size. Targeting the hybrid E / M phenotype, too, had little effect due to the high probability of generating epithelial or mesenchymal daughter cells during hybrid E / M cell division. This behavior would allow for the rapid generation of epithelial and mesenchymal cells in the population, both of which are not targeted by the drug. Co-targeting of epithelial and hybrid E / M cells or that of mesenchymal and hybrid E / M cells did not substantially alter the tumor size either. The un-targeted phenotype took over the population in both cases.

**Fig 8:**
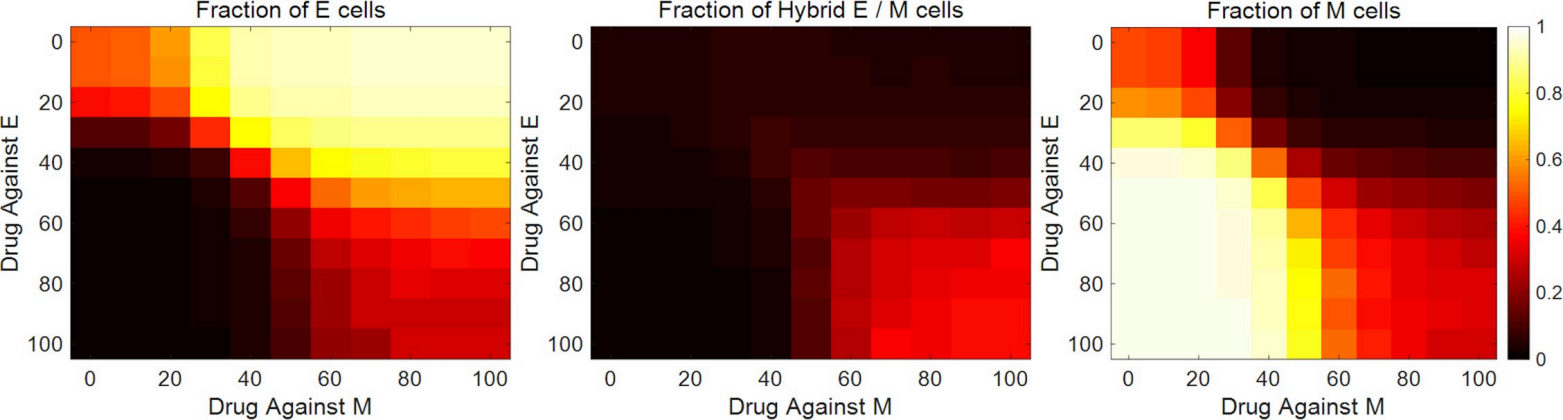
Change in the phenotypic composition of the tumor upon drug treatment. Fractions of different phenotypes in a population upon co-treatment, for *28* days, with varying concentrations of drugs targeting epithelial and mesenchymal cells. On day *0*, all cells in the population were epithelial. Drug concentrations are indicated as *10*^*3*^ units. Here, *η* = *2*.*7*×*10*^*4*^ and the average doubling time of cells was *38*.*0* hours.

A drastic decrease in tumor size was observed upon co-targeting of epithelial and mesenchymal cells (Fig 7). While the resource availability for hybrid E / M cells went up in this regime due to the depletion of epithelial and mesenchymal populations, hybrid E / M cells, with low rates of symmetric self-renewal, quickly generated a population with epithelial and mesenchymal cells. Both these phenotypes were subsequently targeted. This led to a drastic decline in tumor size with greater decline at higher drug dosages. Under this regime of co-targeting epithelial and mesenchymal cells, the phenotypic composition of the tumor cell population was also fundamentally altered—fractions of the three phenotypes in the population became nearly equal (Fig 8). Thus, simultaneous targeting of epithelial and mesenchymal cells, while effective in restricting tumor growth, can bolster phenotypic heterogeneity and increase the fraction of hybrid E / M cells in the population. The hybrid E / M phenotype has been implicated in multiple metastatically aggressive behaviors [30]. Thus, the treatment regime, though effective in restricting tumor growth, will have significant implications for cancer relapse post-treatment.

## Discussion

Multiple studies have characterized the phenotypic heterogeneity in populations of cancer cells [66]. However, the ability of cancer cell populations to retain this heterogeneity over extended periods of time and through multiple generations and passages has remained a mystifying feature, with wide-ranging implications for the design of anti-cancer therapeutic strategies. Here, we have described a model for the generation and maintenance of epithelial-mesenchymal heterogeneity in a population of cancer cells. Our model is based upon the multi-stability of the regulatory circuit driving EMT and MET, and thus connects the dynamical behavior of the regulatory mechanism within each cell to the phenotypic composition of the population. While the model does not capture the rich behavior that may arise from the variable strengths of regulatory interactions in cells in a population, from cell-cell communication, or from regulation of EMT via epigenetic mechanisms [67], it is a useful first step in understanding the generation and maintenance of phenotypic heterogeneity in cancer cells. Targeting the ability of cancer cells to change phenotypes and generate heterogeneous populations has recently been proposed as a therapeutic strategy for combating drug resistance [14]. Our model can be a platform to identify putative therapeutic targets for inhibiting the generation, maintenance, and propagation of phenotypic heterogeneity in populations of cancer cells that exhibit epithelial-mesenchymal plasticity.

A key prediction of our model is that noise in the partitioning of parent cell biomolecules among the daughter cells at the time of cell division is sufficient for the generation of phenotypic heterogeneity in a population of cancer cells. Since the heterogeneity arises from cell division-associated noise, it can be maintained over multiple generations and can be propagated from a small population. This makes it possible for a small population of tumor cells, like that leftover after surgery or after a chemotherapeutic regime, to recreate the full-fledged phenotypic heterogeneity of a mature tumor if conditions that can sustain tumor growth emerge at a later point in time. The hybrid E / M phenotype can quickly generate a mixed population of epithelial and mesenchymal cells. This behavior may contribute to the metastatic aggressiveness widely associated with the hybrid E /M phenotype [30] and may account for the abundance of these cells in aggressive disease subtypes such as inflammatory breast cancer [62]. Further, hybrid E / M cells, when disseminated to a distant organ, can potentially re-create the phenotypic complexity of the primary tumor at the distant organ site, providing a suitable microenvironment for tumor growth in the new tissue. Our model thus highlights how the initial phenotypic composition can affect the temporal population-level behavior of tumor cell populations and the implications of such behavior for cancer metastasis.

Another key prediction of our model is hysteresis in the temporal dynamics of fractions of different phenotypes in a population of cancer cells. Note that the hysteretic behavior reported here is distinct from the behavior observed in typical non-linear systems where the steady state reached by a system can depend on the input history. In fact, in our model, the population of cells does not reach a steady state of fractions of different phenotypes within the time period over which most experimental studies are carried out. Rather, hysteresis is observed in the trajectories displayed by the fractions of different phenotypes over experimental time scales, a behavior that has been observed in multiple cell lines [36]. Our model further predicts that some cancer cells undergo a seemingly irreversible EMT upon transient exposure to an EMT inducer such as TGF-*β*. These cells can retain a mesenchymal phenotype long after the EMT-inducing signal has been withdrawn and do not revert back to an epithelial state during the time period considered in the present study. The time period we have considered is typical of most experimental studies. An irreversible EMT upon chronic exposure to EMT-inducing signals has been reported [19,68]. Our model shows that even transient activation of EMT signaling in cancer cells can generate heterogeneity which will be maintained and propagated long after the exogenous signal has been withdrawn. Such behavior has been confirmed in a recent study which showed that transient exposure to TGF-*β* can be sufficient to generate cells with a mesenchymal phenotype and such cells retain the acquired mesenchymal phenotype for days after TGF-*β* withdrawal [36]. Previous studies have shown how autocrine TGF-*β* signaling [19,69] and epigenetic feedback in EMT regulation [67] can bring about a seemingly irreversible EMT in cancer cells. Further, auto-regulatory control such as the one driving ZEB1 can contribute towards gene expression noise [70] and modulate phenotypic composition of populations [71]. Here, we have demonstrated that noisy partitioning during cell division is another mechanism that can lead to the emergence of such behavior.

Finally, our model provides significant insight into how phenotypically plastic populations will respond to drugs. When cells that can generate cells of other phenotypes are treated with a drug that targets only one phenotype, untargeted cells, generated from the drug-sensitive cells, will take over the population with no significant decline in tumor size. Similar behavior has been described in the context of adaptive cancer therapy wherein the small number of chemotherapy-resistant cells present in the tumor can take over the population once the chemo-sensitive population has been killed off [72]. In our model, under drug activity, cells can switch between drug-sensitive and drug-resistant phenotypes which markedly alters the population response to the drug. Leaving one of the more stable phenotypes untargeted will lead to that phenotype taking over the population with no decline in tumor size. In the context of tumor cells that can exhibit epithelial-mesenchymal plasticity, this is observed in the scenario wherein either epithelial or mesenchymal cells are left untargeted. Co-targeting both these phenotypes resulted in the best therapeutic response with respect to decrease in tumor size. However, even in this regime, a small, highly heterogeneous population is left behind which can quickly regenerate the full-fledged phenotypic heterogeneity of a mature tumor once the drug treatment has been withdrawn. Thus, our model highlights the difficulty in developing effective therapeutic strategies for the heterogenous and plastic disease that is cancer.

While the present study focuses on epithelial-mesenchymal heterogeneity in cancer cells, the model can easily be generalized to describe spontaneous changes in the phenotypic composition of a cancer cell population in multiple scenarios wherein the regulatory mechanism determining cell fate has been characterized. Our model can easily be adapted to describe stem-cell heterogeneity in the tumor microenvironment [24], neuroendocrine plasticity in small cell lung cancer [15], and luminal-basal plasticity in triple-negative breast cancer [14]. Further, the model can be extended to describe spatio-temporal heterogeneity generated via a juxtacrine signaling mechanism such as Notch signaling [40,73] or via paracrine signaling between cancer cells and immune cells [74].

## Methods

### Computer simulation of the model

The dynamics of the regulatory circuit were simulated using ordinary differential equations (ODEs). The mathematical form of the ODEs and the relevant kinetic parameters are described in the S1 Text (Tables A and B). Population dynamics were simulated using Gillespie’s algorithm [75]. The model parameters are listed in the S1 Text. C++ code used to generate the figures presented in the study is available online on GitHub (https://github.com/st35/cancer-EMT-heterogeneity-noise).

## Supporting information

S1 Text Detailed description of development of the noise model and of the simulations carried out in the present study. The file also includes the model parameters used to generate the data shown in the study.
(DOCX)

S1 Fig Growth kinetics of tumor cells for different values of the model parameters. Growth kinetics were unaffected upon varying the noise parameter *η* (left panel). Time taken to reach a steady state in terms of the population size increased upon increasing the average doubling of cells in the population (center panel). Increasing the death rate of cells in the population decreased the population size in the steady state but did not alter the time taken to reach the steady state (right panel). In each case, simulations were started with a population of *500* cells on day *0* and a fixed carrying capacity of *10000* cells. In the left and right panels, average doubling time of cells was *38*.*0* hours. In the center and right panels, *η* = *1*.*0*×*10*^*4*^. The results shown here were obtained by averaging over *16* distinct simulation runs. Error bars indicate the standard deviation calculated over these runs.
(TIF)

S2 Fig Effect of death rate of cells on the kinetics of change in the phenotypic composition of a population. Here, *η* = *1*.*0*×*10*^*4*^ and the average doubling time of cells in the population was *38*.*0* hours. The results shown here were obtained by averaging over *16* distinct simulation runs. Error bars indicate the standard deviation calculated over these runs. The carrying capacity in our model is fixed. Therefore, when the death rate of cells is low, there will be few cell division events once the population size has reached a steady state. Since the phenotypic composition of the population changes mainly due to a daughter cell acquiring a phenotype different from that of the parent cell, the phenotypic composition of the population will not change much with time at low cell death rates (blue curves in the three panels). As the death rate increases, cell division events can take place in the steady state to replace the dead cells. As a result, the phenotypic composition changes at a faster rate (orange curves in the three panels). However, changing the death rate of cells by two orders of magnitude has limited effect on the kinetics of change in the phenotypic composition of the population.
(TIF)

S3 Fig Heterogeneity in the phenotypic composition of populations of cancer cells that can exhibit EMP. (A) Fractions of epithelial, hybrid E / M, and mesenchymal cells at different time points in populations that had distinct phenotypic compositions on day *0*. Rows indicate populations with different initial phenotypic compositions and columns indicate different time points. Here, *η* = *2*.*7*×*10*^*4*^ and the average doubling time of cells was *38*.*0* hours. (B) EMT scores for cell lines commonly used in experiments to investigate epithelial-mesenchymal plasticity. Scores were calculated using gene expression profiles of cell lines from studies wherein the expression had been profiled in the untreated (or control) regime. A score below *0*.*5* indicates an epithelial phenotype while a score above *1*.*5* indicates a mesenchymal phenotype. A score between *0*.*5* and *1*.*5* indicates a hybrid E / M phenotype. All gene expression profiles were obtained from public databases (see Table C in S1 Text for a list of all the datasets). Though scores for each cell line were calculated using only those gene expression profiles that were obtained in the untreated regime (i.e., cells not exposed to any reagent that may promote or inhibit EMT / MET), there is notable variation in scores for a given cell line across independent studies.
(TIF)

S4 Fig Model dynamics under a two-state model of EMP regulation. (A) EMP regulatory circuit whose behavior was analyzed by Celià -Terrassa *et al*. (left panel) and bifurcation diagram for this regulatory circuit (right panel). The circuit clearly exhibits two types of stable steady states—epithelial, characterized by high *CDH1* expression (shown in green), and mesenchymal, characterized by low *CDH1* expression (shown in orange). (B) Phenotypic composition over time of populations of cells as predicted by combining our model of partitioning noise during cell division with the model of EMP regulation analyzed by Celià -Terrassa *et al*. Since TGF-*β* is the key driver of EMT in this model, only the noise in the partitioning of this circuit component was considered. Different colors in panels of (B) indicate the behavior for different values of the noise parameter *η*. All cells in these simulations had an average doubling time of *38*.*0* hours. Mathematical equations and the parameters governing behavior of the network in (A) were taken from Celià -Terrassa *et al*. The results shown here were obtained by averaging over *16* distinct simulation runs. Error bars indicate the standard deviation calculated over these runs.
(TIF)

S5 Fig Model dynamics under a four-state model of EMP regulation. (A) EMP regulatory circuit whose behavior was analyzed by Hong *et al*. (left panel) and the bifurcation diagram for this regulatory circuit (right panel). The network exhibits four types of stable steady states. The four types can be mapped to four distinct EMP-associated phenotypes. (B) Phenotypic composition over time of populations of cells as predicted by combining our model of partitioning noise during cell division with the model of EMP regulation analyzed by Hong *et al*. Since external TGF-*β* is the key driver of EMT in this model, only the noise in the partitioning of this circuit component was considered. Different colors in the panels of (B) indicate the behavior for different values of the noise parameter *η*. All cells in these simulations had an average doubling time of *38*.*0* hours. The results shown here were obtained by averaging over *16* distinct simulation runs. Error bars indicate the standard deviation calculated over these runs. Panels in (A) are reproduced from Hong *et al*. under a Creative Commons Attribution License. Mathematical equations and the parameters governing behavior of the network in (A) were taken from Hong *et al*.
(TIF)

S6 Fig (A) Hybrid E / M cells exhibit an intermediate level of miR-34a expression as compared to epithelial cells (high miR-34a expression) and mesenchymal cells (low miR-34a expression). (B) Root mean square deviation (RMSD) of model predictions from experimental data for murine prostate cancer cells obtained from Ruscetti *et al*. RMSD was calculated by pooling fractions of the three phenotypes at different time points in the three cases—when starting with a population of only epithelial cells, when starting with a population of only hybrid E / M cells, and when starting with a population of only mesenchymal cells. A lower RMSD was obtained in the model with asymmetric distribution of miR-34a among the daughter cells during the division of hybrid E / M cells. *I*_*sig*_ concentration above a threshold leads to a mesenchymal phenotype. Also, *I*_*sig*_ concentration cannot fall below *0*.*0*. Thus, at very high values of *η*, the fraction of mesenchymal cells in the population will increase rapidly leading to large deviation from experimental data at high *η*. At low *η*, the probability of a daughter cell acquiring a phenotype different from that of the parent cell will be very low (Fig 2(A)). Thus, at very low values of *η*, the model will not be able to capture the plasticity of hybrid E / M populations. Large deviations from experimental behavior at low and high *η* account for the non-monotonic nature of the curves in this figure.
(TIF)

S7 FigFitting to experimental data in the presence of multiple inputs to the core EMP regulatory circuit.These include signals promoting EMT and those inhibiting EMT. The core EMP regulatory circuit with *3* EMT-inducing and *2* EMT-inhibiting signals is shown in the left panel. In the right panel, we show the root mean square deviation (RMSD) of model predictions from experimental data for murine prostate cancer cells obtained from Ruscetti *et al*. The model predictions were obtained using the same value of the noise parameter *η* for each input signal. In the presence of multiple EMT-inducing and EMT-inhibiting signals, a good fit to experimental data can be obtained at a much lower value of the noise parameter *η*. Once again, a lower RMSD is obtained when miR-34a is asymmetrically distributed among the daughter cells during the division of hybrid E / M cells. The kinetic parameters governing the regulation of *SNAI1* by *I*_*sig*_ was kept the same for each input to the regulatory circuit. Only $\lambda_{I}^{m_{S}}$, the fold change in the production rate of SNAI1 mRNA in response to the input, differed for the activating and inhibitory inputs. For activating inputs, $\lambda_{I}^{m_{S}}=10.0$, and for inhibitory inputs, $\lambda_{I}^{m_{S}}=0.1$.(TIF)

S8 Fig Fitting to experimental data by varying the growth rate of cells in the population. (A) Root mean square deviation (RMSD) of model predictions from experimental data for murine prostate cancer cells obtained from Ruscetti *et al*., plotted as a function of the average doubling time of cells in the population. Here, *η* = *2*.*0*×*10*^*4*^. Similar to the RMSD curves shown in S6 Fig. (B) and S7 Fig (right panel), the RMSD varies non-monotonically. A lower RMSD is obtained when miR-34a is asymmetrically partitioned among the daughter cells during the division of a hybrid E / M cell. (B) and (C) Fractions of different phenotypes assessed at different time points during a two-week period. Model predictions (solid curves) represent the best fit obtained by varying the average doubling time of cells while keeping the value of the noise parameter fixed (*η* = *2*.*0*×*10*^*4*^). (B) shows the model predictions when there is no asymmetric distribution of miR-34a during cell division (best fit obtained for an average doubling time of *18*.*0* hours) while (C) shows the model predictions when miR-34a is asymmetrically distributed among the daughter cells during the division of hybrid E / M cells (best fit obtained for an average doubling time of *21*.*0* hours). Dotted curves represent the fractions of different phenotypes as re-plotted from Ruscetti *et al*. Model predictions shown in (B) and (C) were obtained by averaging over *16* distinct simulation runs. Error bars indicate the standard deviation calculated over these runs.
(TIF)

S9 Fig Effect of different growth phenotypes that may be exhibited by hybrid E / M cells on model behavior. (A) Behavior when hybrid E / M cells exhibit a hyper proliferative phenotype (average doubling time of *8*.*0* hours). (B) Behavior when hybrid E / M cells exhibit a growth arrested phenotype (average doubling time of *192*.*0* hours or *8* days). (C) Behavior when hybrid E / M cells exhibit mixed growth phenotype—each hybrid E / M cell was either hyper proliferative (average doubling time of *8*.*0* hours) or growth arrested (average doubling time of *192*.*0* hours or *8* days), with equal probabilities. Dashed curves in each plot indicate the behavior when all cells (epithelial, mesenchymal, or hybrid E / M) have the same growth rates (average doubling time of *38*.*0* hours). In each simulation, *η* = *2*.*5*×*10*^*4*^ with asymmetric partitioning of miR-34a among the daughter cells during hybrid E / M cell division. All results were obtained by averaging over *16* distinct simulation runs. Error bars indicate the standard deviation calculated over these runs. As expected, behavior of epithelial and mesenchymal populations is unaffected by the growth phenotype of hybrid E / M cells within the time period for which the population dynamics were simulated (left and right panels in (A), (B), and (C)). When hybrid E / M cells were hyper-proliferative, the rate of decline in the fraction of these cells in a population that was purely hybrid E / M on day *0* increased ((A); center panel). In contrast, the rate of decline was lowered when hybrid E / M cells exhibited a growth arrested phenotype ((B); center panel). This behavior is a consequence of the high probability of generation of a non-hybrid E / M daughter cell when a hybrid E / M cell divides (Fig 2(A); center panel). Higher growth rate of hybrid E / M cells would lead to epithelial or mesenchymal daughter cells being generated at a faster rate at the expense of hybrid E / M parent cells, resulting in a faster decrease in the fraction of hybrid E / M cells in the population. This decrease is slow when hybrid E / M cells divide at a slower rate ((B); center panel). No non-trivial behavior was observed when hybrid E / M cells exhibit a mixed growth phenotype—the rate of decline in the fraction of hybrid E / M cells was somewhere between the rates of decline in the hyper proliferative hybrid E / M and growth arrested hybrid E / M cases ((C); center panel).
(TIF)

S10 Fig Effect of varying growth rates of different cell types on the phenotypic composition of a population of epithelial cells. In each panel, the color indicates the fraction in population of a given phenotype—epithelial (green), hybrid E / M (orange), and mesenchymal (purple) while the line type indicates the behavior for different growth rates of epithelial cells—*8*.*0* hours (dotted with triangular markers), *24*.*0* hours (solid with circular markers), and *192*.*0* hours (dashed with square markers). In each simulation, *η* = *2*.*5*×*10*^*4*^ with asymmetric partitioning of miR-34a among the daughter cells during hybrid E / M cell division. All results were obtained by averaging over *16* distinct simulation runs. Error bars indicate the standard deviation calculated over these runs.
(TIF)

S11 Fig Effect of varying growth rates of different cell types on the phenotypic composition of a population of hybrid E / M cells. In each panel, the color indicates the fraction in population of a given phenotype—epithelial (green), hybrid E / M (orange), and mesenchymal (purple) while the line type indicates the behavior for different growth rates of epithelial cells—*8*.*0* hours (dotted with triangular markers), *24*.*0* hours (solid with circular markers), and *192*.*0* hours (dashed with square markers). In each simulation, *η* = *2*.*5*×*10*^*4*^ with asymmetric partitioning of miR-34a among the daughter cells during hybrid E / M cell division. All results were obtained by averaging over *16* distinct simulation runs. Error bars indicate the standard deviation calculated over these runs.
(TIF)

S12 Fig Effect of varying growth rates of different cell types on the phenotypic composition of a population of mesenchymal cells. In each panel, the color indicates the fraction in population of a given phenotype—epithelial (green), hybrid E / M (orange), and mesenchymal (purple) while the line type indicates the behavior for different growth rates of epithelial cells—*8*.*0* hours (dotted with triangular markers), *24*.*0* hours (solid with circular markers), and *192*.*0* hours (dashed with square markers). In each simulation, *η* = *2*.*5*×*10*^*4*^ with asymmetric partitioning of miR-34a among the daughter cells during hybrid E / M cell division. All results were obtained by averaging over *16* distinct simulation runs. Error bars indicate the standard deviation calculated over these runs.
(TIF)

S13 Fig Effect on model behavior of noise in partitioning of species other than *I*_*sig*_. Solid curves indicate the fractions of different phenotypes when noise in the partitioning of all network species is incorporated into the model (CV = *0*.*25* for all species other than *I*_*sig*_). Dashed curves indicate the behavior when the model only includes noise in the partitioning of *I*_*sig*_. In each simulation, *η* = *2*.*5*×*10*^*4*^ (here, this noise parameter only determines the noise in the partitioning of *I*_*sig*_). miR-34a was asymmetrically partitioned among the daughter cells during hybrid E / M cell division. All results were obtained by averaging over *16* distinct simulation runs. Error bars indicate the standard deviation calculated over these runs.
(TIF)

S14 Fig Effect on model behavior if, during hybrid E / M cell division, one daughter cell gets no miR-34a while the other daughter cell gets twice the concentration of miR-34a in the parent cell. Solid curves indicate the fractions of different phenotypes when, during hybrid E / M cell division, one daughter cells gets no miR-34a while the other gets twice the concentration of miR-34a in the parent cell. Here, the assumption is that during hybrid E / M cell division, all of parent cell miR-34a (note that miR-34a copy number in the parent cell gets approximately doubled right before the parent cell divides) is actively deposited into one of the daughter cells. Dashed curves indicate the behavior when during hybrid E / M cell division, the concentration of miR-34a in one of the daughter cells is set to zero while the concentration of miR-34a in the other daughter cell is kept the same as the concentration of miR-34a in the parent cell. In this case, the assumption is that while parent cell miR-34a is equally divided among the daughter cells during the division of a hybrid E / M cell, in one of the daughter cells, an active process rapidly degrades miR-34a and brings its concentration to zero. In each simulation, *η* = *2*.*5*×*10*^*4*^. All results were obtained by averaging over *16* distinct simulation runs. Error bars indicate the standard deviation calculated over these runs.
(TIF)

S15 Fig Long term behavior of the population after the EMT-inducing signal has been withdrawn. (A) Fraction of epithelial, mesenchymal, and hybrid E / M cells shown over a period of *150* days in the population probed in Fig 4. The black dashed line indicates day *20* after which no addition or subtraction of *I*_*sig*_ dosages was administered. Results shown were obtained by averaging over *16* distinct simulation runs. Error bars indicate the standard deviation calculated over *16* independent runs. (B) Distribution of *I*_*sig*_ concentration in cells in the population at different time points during the simulation run. Here, *η* = *2*.*7*×*10*^*4*^ and the average doubling time of cells was *38*.*0* hours. As shown in (B), at the end of the *20*-day period, most cells in the population either have *I*_*sig*_ concentration close to *0*.*0* or very high *I*_*sig*_ concentration. Few cells have *I*_*sig*_ concentration in the region of tri-stable dynamics of the EMP regulatory circuit (Fig 1(B)). Since cells far away from the tri-stable region, which form the bulk of the population on day *20*, are highly unlikely to generate a daughter cell with a phenotype different from that of the parent cell, the fractions of cells of different phenotypes in the population do not change much for nearly a month after day *20* when the external addition or withdrawal of *I*_*sig*_ dosages was stopped. The population takes a long time to recover from the clustering of *I*_*sig*_ concentrations away from the tri-stable region. However, noise in the partitioning of *I*_*sig*_ among the daughter cells during cell division eventually drives the *I*_*sig*_ concentration in cells back into the tri-stable region and around day *60*, we once again start observing the typical dynamics—decrease in the fraction of epithelial cells and increase in the fraction of mesenchymal cells in the population. A small sub-population of hybrid E / M cells is continuously maintained.
(TIF)

S16 Fig Same as Fig 4 but with asymmetric distribution of miR-34a among the daughter cells during hybrid E / M cell division. Here, *η* = *2*.*5*×*10*^*4*^ which had given the best fit to experimental data from Ruscetti *et al*. when incorporating asymmetric distribution of miR-34a among the daughter cells during hybrid E / M cell division into our model.
(TIF)

S17 Fig Same as Fig 5 but with asymmetric distribution of miR-34a among the daughter cells during hybrid E / M cell division. Here, in (B) and (C), *η* = *2*.*5*×*10*^*4*^ which had given the best fit to experimental data from Ruscetti *et al*. when incorporating asymmetric distribution of miR-34a among the daughter cells during hybrid E / M cell division into our model.
(TIF)

S18 Fig Same as Fig 6 but with asymmetric distribution of miR-34a among the daughter cells during hybrid E / M cell division. Here, *η* = *2*.*5*×*10*^*4*^ which had given the best fit to experimental data from Ruscetti *et al*. when incorporating asymmetric distribution of miR-34a among the daughter cells during hybrid E / M cell division into our model.
(TIF)

S19 Fig Same as Fig 7 but with asymmetric distribution of miR-34a among the daughter cells during hybrid E / M cell division. Here, *η* = *2*.*5*×*10*^*4*^ which had given the best fit to experimental data from Ruscetti *et al*. when incorporating asymmetric distribution of miR-34a among the daughter cells during hybrid E / M cell division into our model.
(TIF)

S20 Fig Same as Fig 8 but with asymmetric distribution of miR-34a among the daughter cells during hybrid E / M cell division. Here, *η* = *2*.*5*×*10*^*4*^ which had given the best fit to experimental data from Ruscetti *et al*. when incorporating asymmetric distribution of miR-34a among the daughter cells during hybrid E / M cell division into our model.
(TIF)
