# Supplementary figures and images for: A mechanism for epithelial-mesenchymal heterogeneity in a population of cancer cells
Source: PLoS Comput Biol. 2020 Feb 10;16(2):e1007619. doi: 10.1371/journal.pcbi.1007619 (PMC7034928; doi:10.1371/journal.pcbi.1007619)

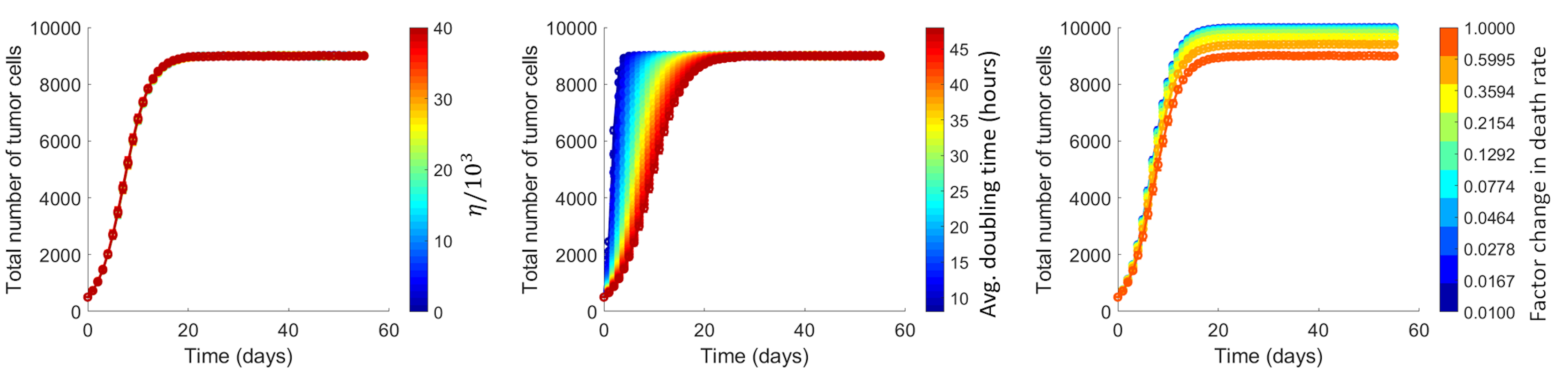

Supplement: S1 Fig — Growth kinetics were unaffected upon varying the noise parameter η (left panel). Time taken to reach a steady state in terms of the population size increased upon increasing the average doubling of cells in the population (center panel). Increasing the death rate of cells in the population decreased the population size in the steady state but did not alter the time taken to reach the steady state (right panel). In each case, simulations were started with a population of 500 cells on day 0 and a fixed carrying capacity of 10000 cells. In the left and right panels, average doubling time of cells was 38.0 hours. In the center and right panels, η = 1.0×10^4^. The results shown here were obtained by averaging over 16 distinct simulation runs. Error bars indicate the standard deviation calculated over these runs. (TIF) [file pcbi.1007619.s002.tif]

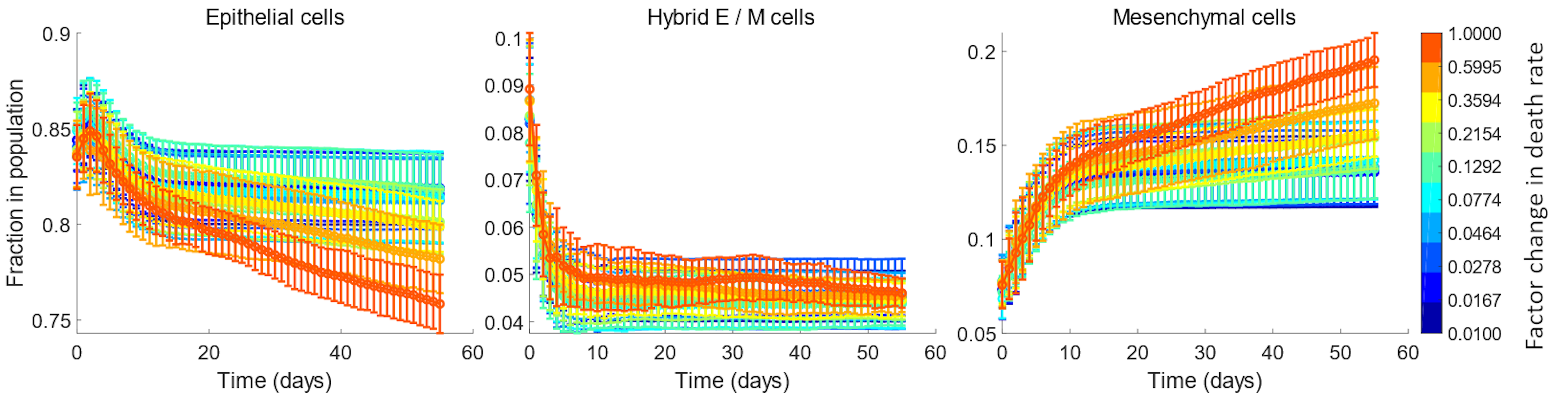

Supplement: S2 Fig — Here, η = 1.0×10^4^ and the average doubling time of cells in the population was 38.0 hours. The results shown here were obtained by averaging over 16 distinct simulation runs. Error bars indicate the standard deviation calculated over these runs. The carrying capacity in our model is fixed. Therefore, when the death rate of cells is low, there will be few cell division events once the population size has reached a steady state. Since the phenotypic composition of the population changes mainly due to a daughter cell acquiring a phenotype different from that of the parent cell, the phenotypic composition of the population will not change much with time at low cell death rates (blue curves in the three panels). As the death rate increases, cell division events can take place in the steady state to replace the dead cells. As a result, the phenotypic composition changes at a faster rate (orange curves in the three panels). However, changing the death rate of cells by two orders of magnitude has limited effect on the kinetics of change in the phenotypic composition of the population. (TIF) [file pcbi.1007619.s003.tif]

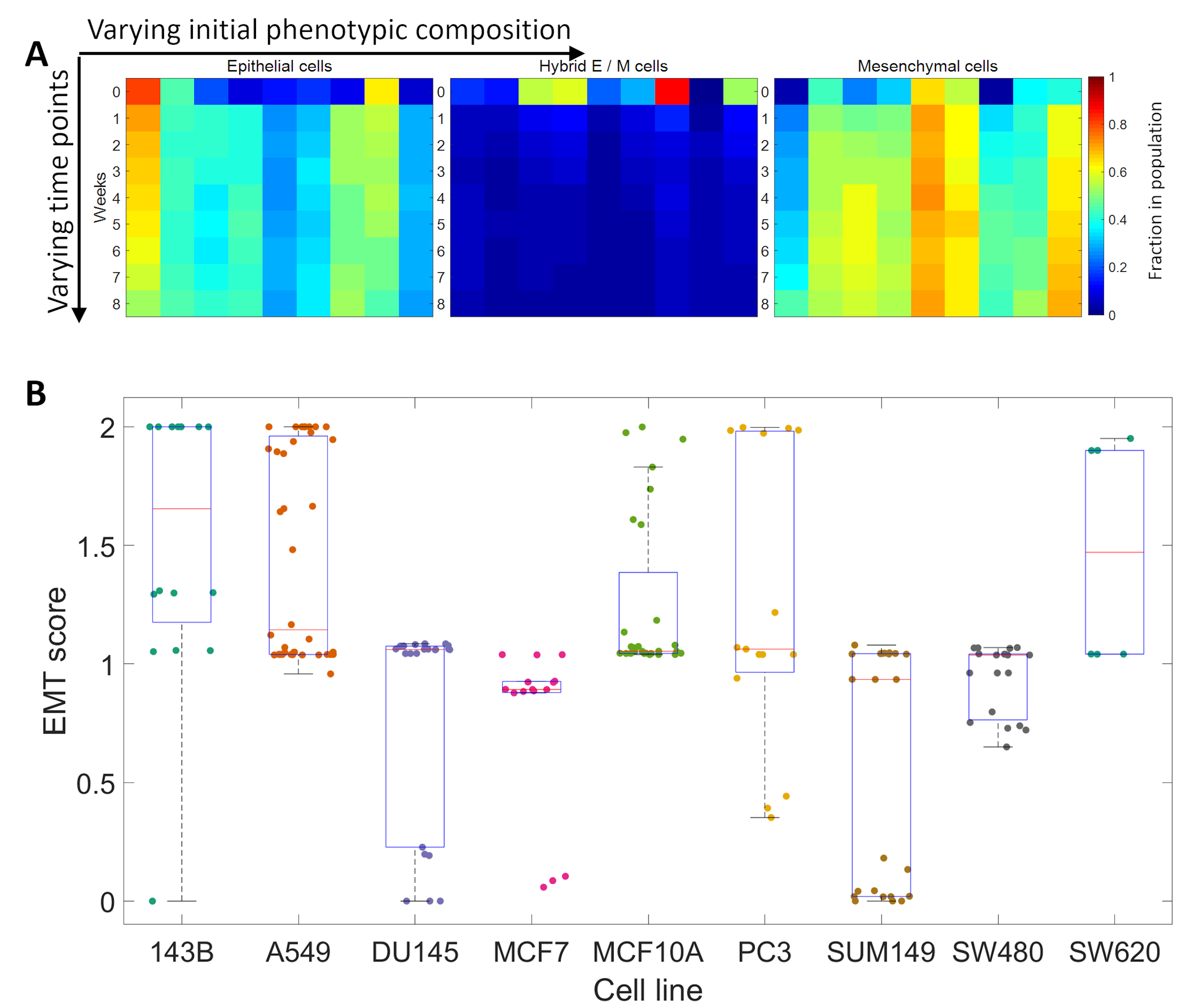

Supplement: S3 Fig — (A) Fractions of epithelial, hybrid E / M, and mesenchymal cells at different time points in populations that had distinct phenotypic compositions on day 0. Rows indicate populations with different initial phenotypic compositions and columns indicate different time points. Here, η = 2.7×10^4^ and the average doubling time of cells was 38.0 hours. (B) EMT scores for cell lines commonly used in experiments to investigate epithelial-mesenchymal plasticity. Scores were calculated using gene expression profiles of cell lines from studies wherein the expression had been profiled in the untreated (or control) regime. A score below 0.5 indicates an epithelial phenotype while a score above 1.5 indicates a mesenchymal phenotype. A score between 0.5 and 1.5 indicates a hybrid E / M phenotype. All gene expression profiles were obtained from public databases (see Table C in S1 Text for a list of all the datasets). Though scores for each cell line were calculated using only those gene expression profiles that were obtained in the untreated regime (i.e., cells not exposed to any reagent that may promote or inhibit EMT / MET), there is notable variation in scores for a given cell line across independent studies. (TIF) [file pcbi.1007619.s004.tif]

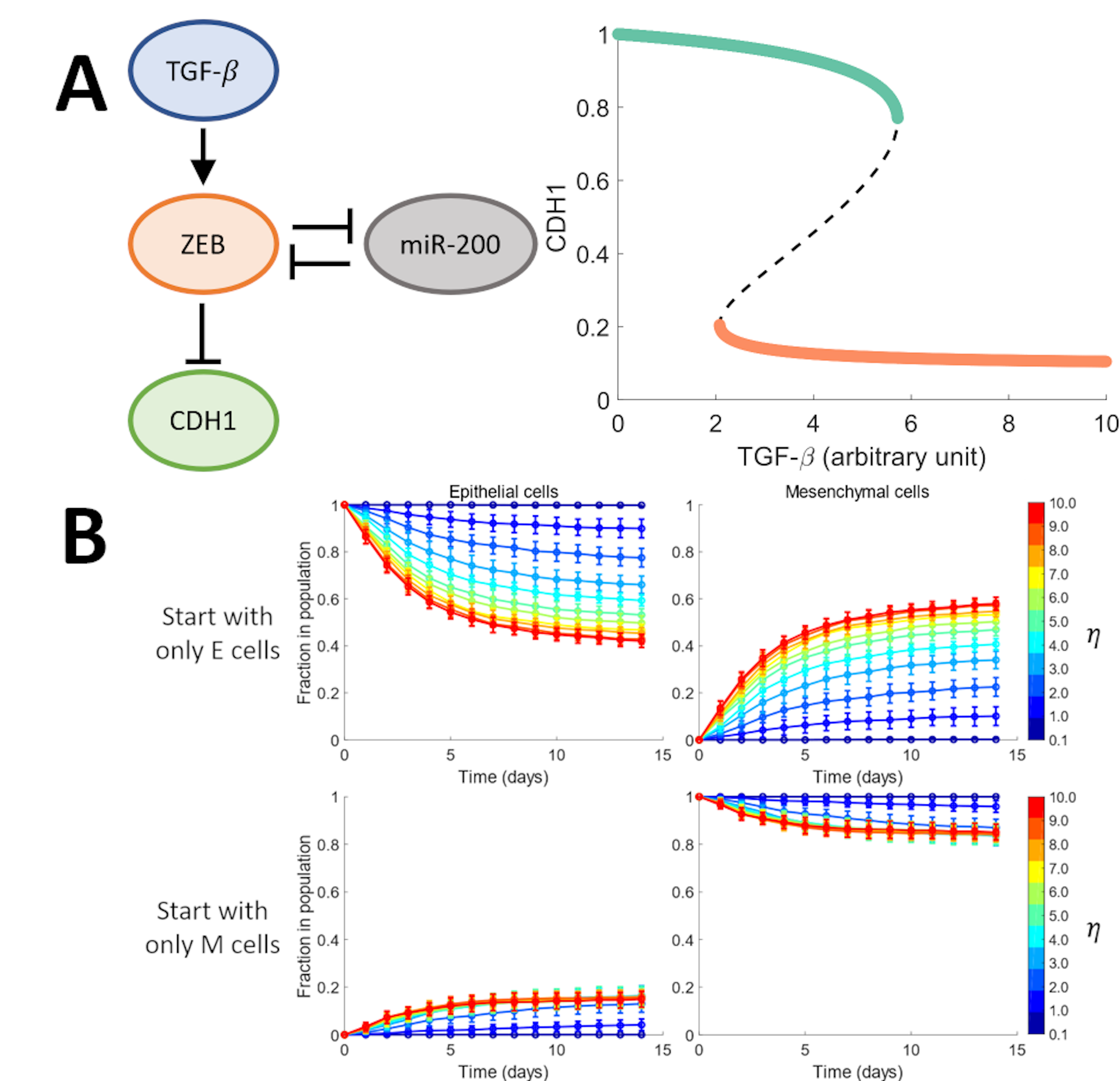

Supplement: S4 Fig — (A) EMP regulatory circuit whose behavior was analyzed by Celià -Terrassa et al. (left panel) and bifurcation diagram for this regulatory circuit (right panel). The circuit clearly exhibits two types of stable steady states—epithelial, characterized by high CDH1 expression (shown in green), and mesenchymal, characterized by low CDH1 expression (shown in orange). (B) Phenotypic composition over time of populations of cells as predicted by combining our model of partitioning noise during cell division with the model of EMP regulation analyzed by Celià -Terrassa et al. Since TGF-β is the key driver of EMT in this model, only the noise in the partitioning of this circuit component was considered. Different colors in panels of (B) indicate the behavior for different values of the noise parameter η. All cells in these simulations had an average doubling time of 38.0 hours. Mathematical equations and the parameters governing behavior of the network in (A) were taken from Celià -Terrassa et al. The results shown here were obtained by averaging over 16 distinct simulation runs. Error bars indicate the standard deviation calculated over these runs. (TIF) [file pcbi.1007619.s005.tif]

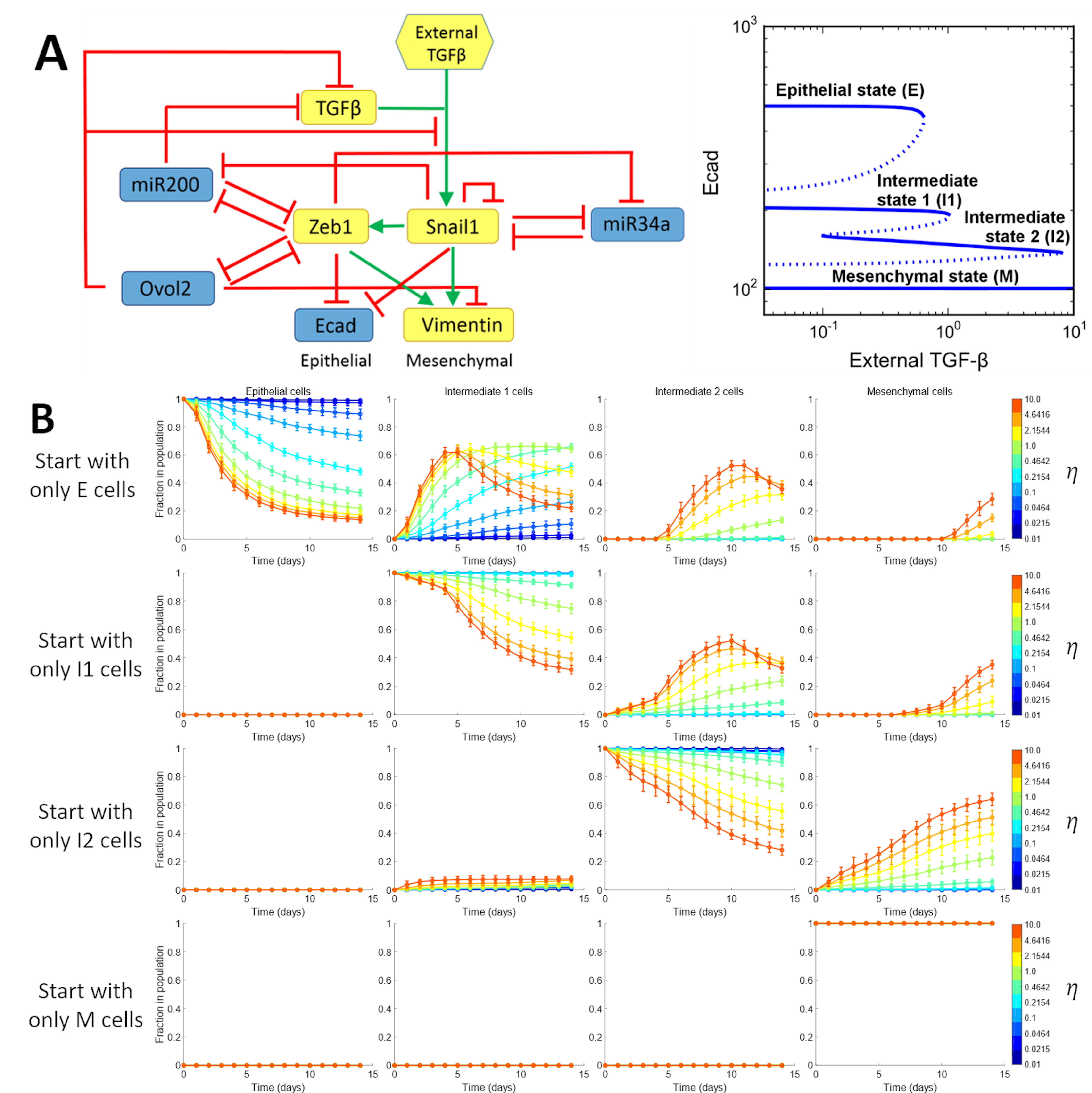

Supplement: S5 Fig — (A) EMP regulatory circuit whose behavior was analyzed by Hong et al. (left panel) and the bifurcation diagram for this regulatory circuit (right panel). The network exhibits four types of stable steady states. The four types can be mapped to four distinct EMP-associated phenotypes. (B) Phenotypic composition over time of populations of cells as predicted by combining our model of partitioning noise during cell division with the model of EMP regulation analyzed by Hong et al. Since external TGF-β is the key driver of EMT in this model, only the noise in the partitioning of this circuit component was considered. Different colors in the panels of (B) indicate the behavior for different values of the noise parameter η. All cells in these simulations had an average doubling time of 38.0 hours. The results shown here were obtained by averaging over 16 distinct simulation runs. Error bars indicate the standard deviation calculated over these runs. Panels in (A) are reproduced from Hong et al. under a Creative Commons Attribution License. Mathematical equations and the parameters governing behavior of the network in (A) were taken from Hong et al. (TIF) [file pcbi.1007619.s006.tif]

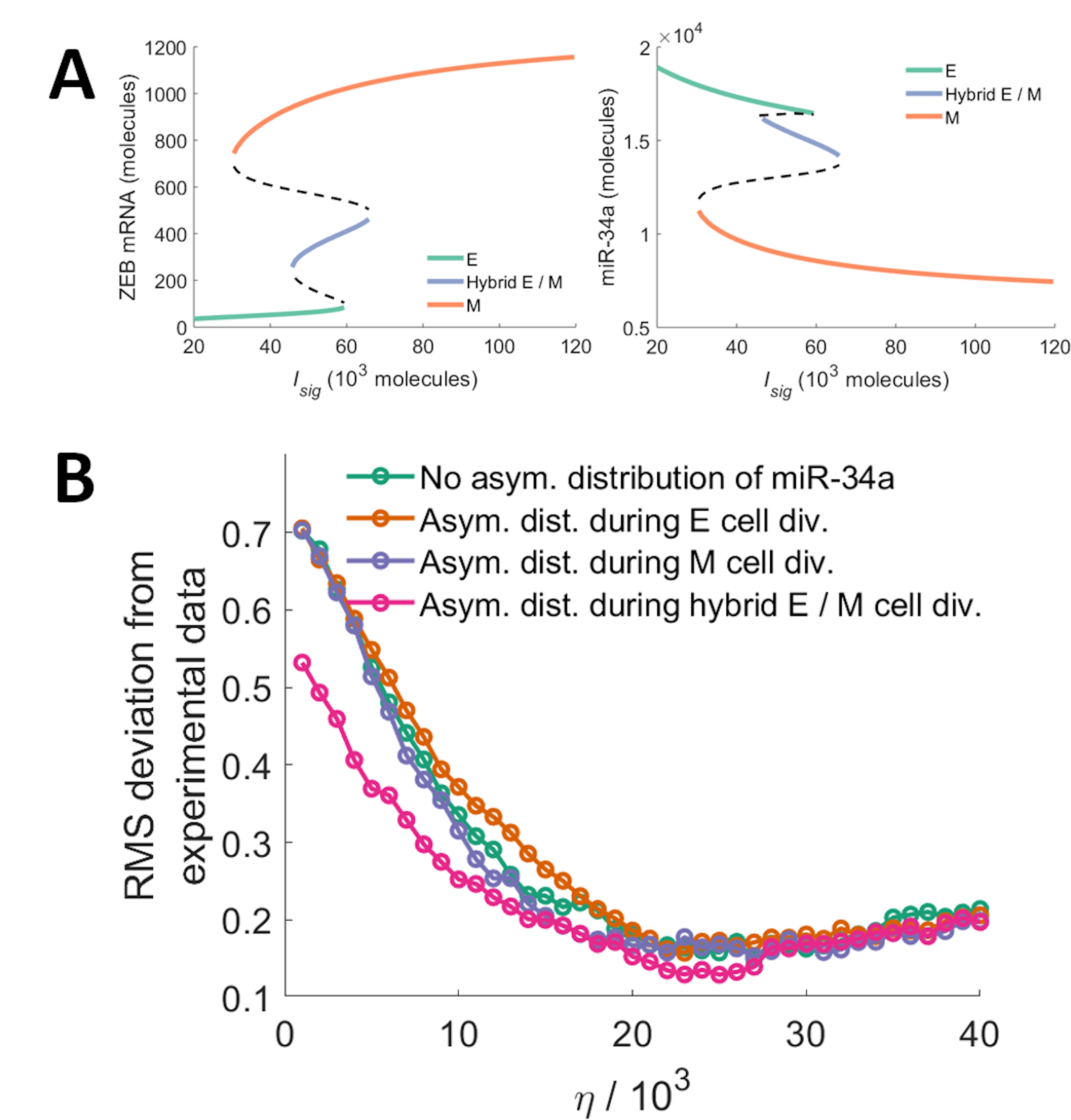

Supplement: S6 Fig — (A) Hybrid E / M cells exhibit an intermediate level of miR-34a expression as compared to epithelial cells (high miR-34a expression) and mesenchymal cells (low miR-34a expression). (B) Root mean square deviation (RMSD) of model predictions from experimental data for murine prostate cancer cells obtained from Ruscetti et al. RMSD was calculated by pooling fractions of the three phenotypes at different time points in the three cases—when starting with a population of only epithelial cells, when starting with a population of only hybrid E / M cells, and when starting with a population of only mesenchymal cells. A lower RMSD was obtained in the model with asymmetric distribution of miR-34a among the daughter cells during the division of hybrid E / M cells. Isig concentration above a threshold leads to a mesenchymal phenotype. Also, Isig concentration cannot fall below 0.0. Thus, at very high values of η, the fraction of mesenchymal cells in the population will increase rapidly leading to large deviation from experimental data at high η. At low η, the probability of a daughter cell acquiring a phenotype different from that of the parent cell will be very low (Fig 2(A)). Thus, at very low values of η, the model will not be able to capture the plasticity of hybrid E / M populations. Large deviations from experimental behavior at low and high η account for the non-monotonic nature of the curves in this figure. (TIF) [file pcbi.1007619.s007.tif]

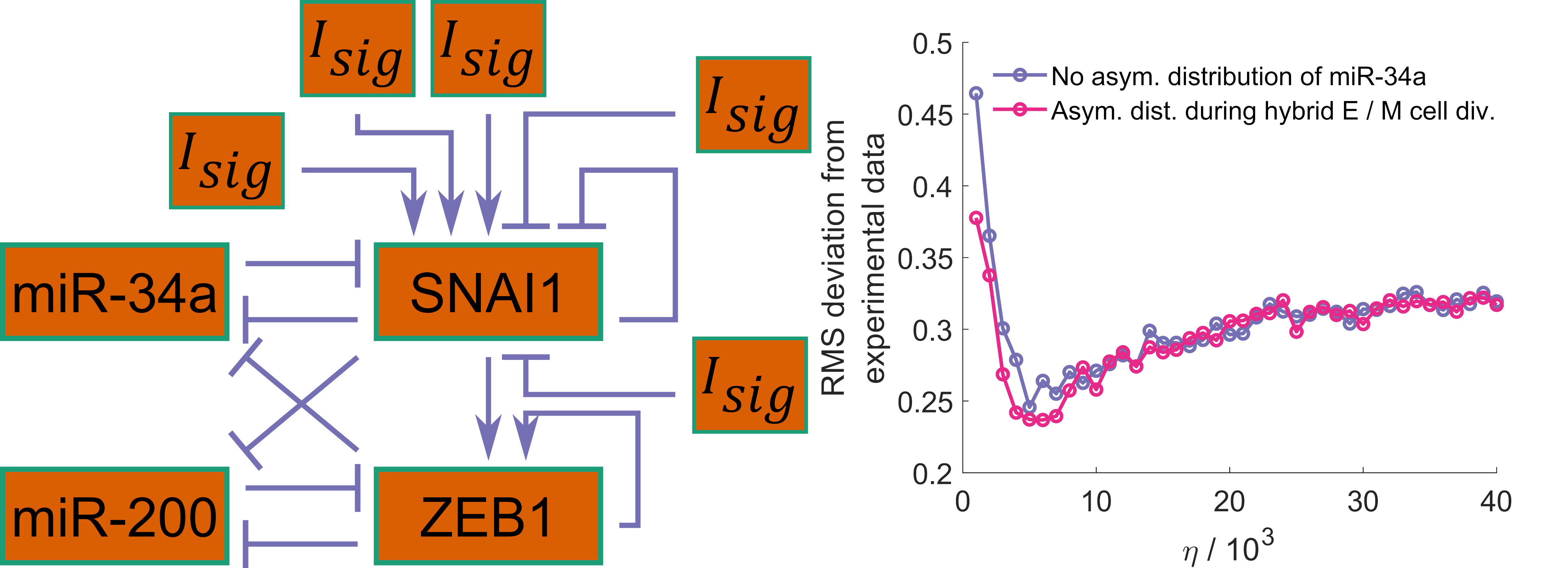

Supplement: S7 Fig — These include signals promoting EMT and those inhibiting EMT. The core EMP regulatory circuit with 3 EMT-inducing and 2 EMT-inhibiting signals is shown in the left panel. In the right panel, we show the root mean square deviation (RMSD) of model predictions from experimental data for murine prostate cancer cells obtained from Ruscetti et al. The model predictions were obtained using the same value of the noise parameter η for each input signal. In the presence of multiple EMT-inducing and EMT-inhibiting signals, a good fit to experimental data can be obtained at a much lower value of the noise parameter η. Once again, a lower RMSD is obtained when miR-34a is asymmetrically distributed among the daughter cells during the division of hybrid E / M cells. The kinetic parameters governing the regulation of SNAI1 by Isig was kept the same for each input to the regulatory circuit. Only λImS, the fold change in the production rate of SNAI1 mRNA in response to the input, differed for the activating and inhibitory inputs. For activating inputs, λImS=10.0, and for inhibitory inputs, λImS=0.1. (TIF) [file pcbi.1007619.s008.tif]

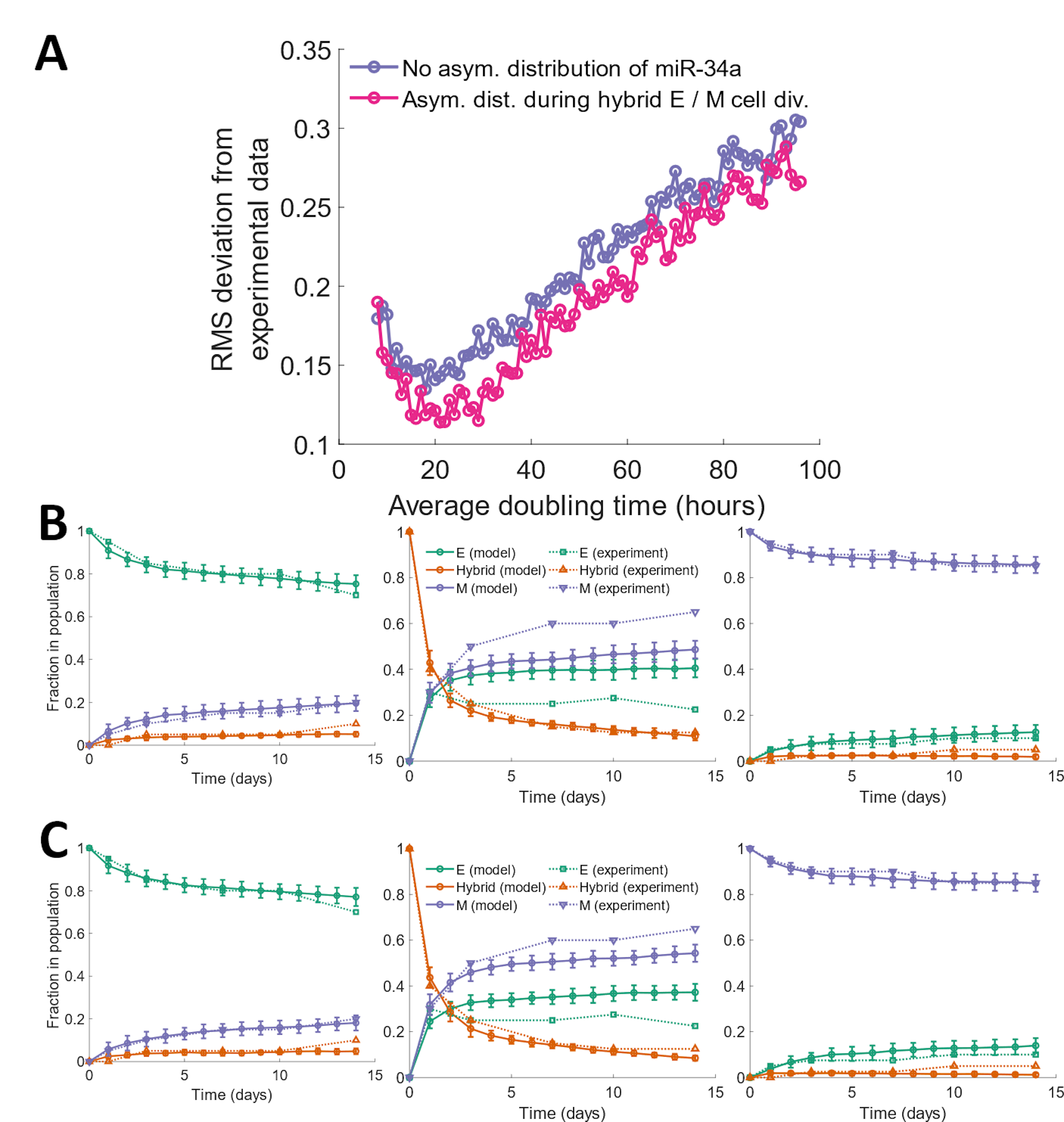

Supplement: S8 Fig — (A) Root mean square deviation (RMSD) of model predictions from experimental data for murine prostate cancer cells obtained from Ruscetti et al., plotted as a function of the average doubling time of cells in the population. Here, η = 2.0×10^4^. Similar to the RMSD curves shown in S6 Fig. (B) and S7 Fig (right panel), the RMSD varies non-monotonically. A lower RMSD is obtained when miR-34a is asymmetrically partitioned among the daughter cells during the division of a hybrid E / M cell. (B) and (C) Fractions of different phenotypes assessed at different time points during a two-week period. Model predictions (solid curves) represent the best fit obtained by varying the average doubling time of cells while keeping the value of the noise parameter fixed (η = 2.0×10^4^). (B) shows the model predictions when there is no asymmetric distribution of miR-34a during cell division (best fit obtained for an average doubling time of 18.0 hours) while (C) shows the model predictions when miR-34a is asymmetrically distributed among the daughter cells during the division of hybrid E / M cells (best fit obtained for an average doubling time of 21.0 hours). Dotted curves represent the fractions of different phenotypes as re-plotted from Ruscetti et al. Model predictions shown in (B) and (C) were obtained by averaging over 16 distinct simulation runs. Error bars indicate the standard deviation calculated over these runs. (TIF) [file pcbi.1007619.s009.tif]

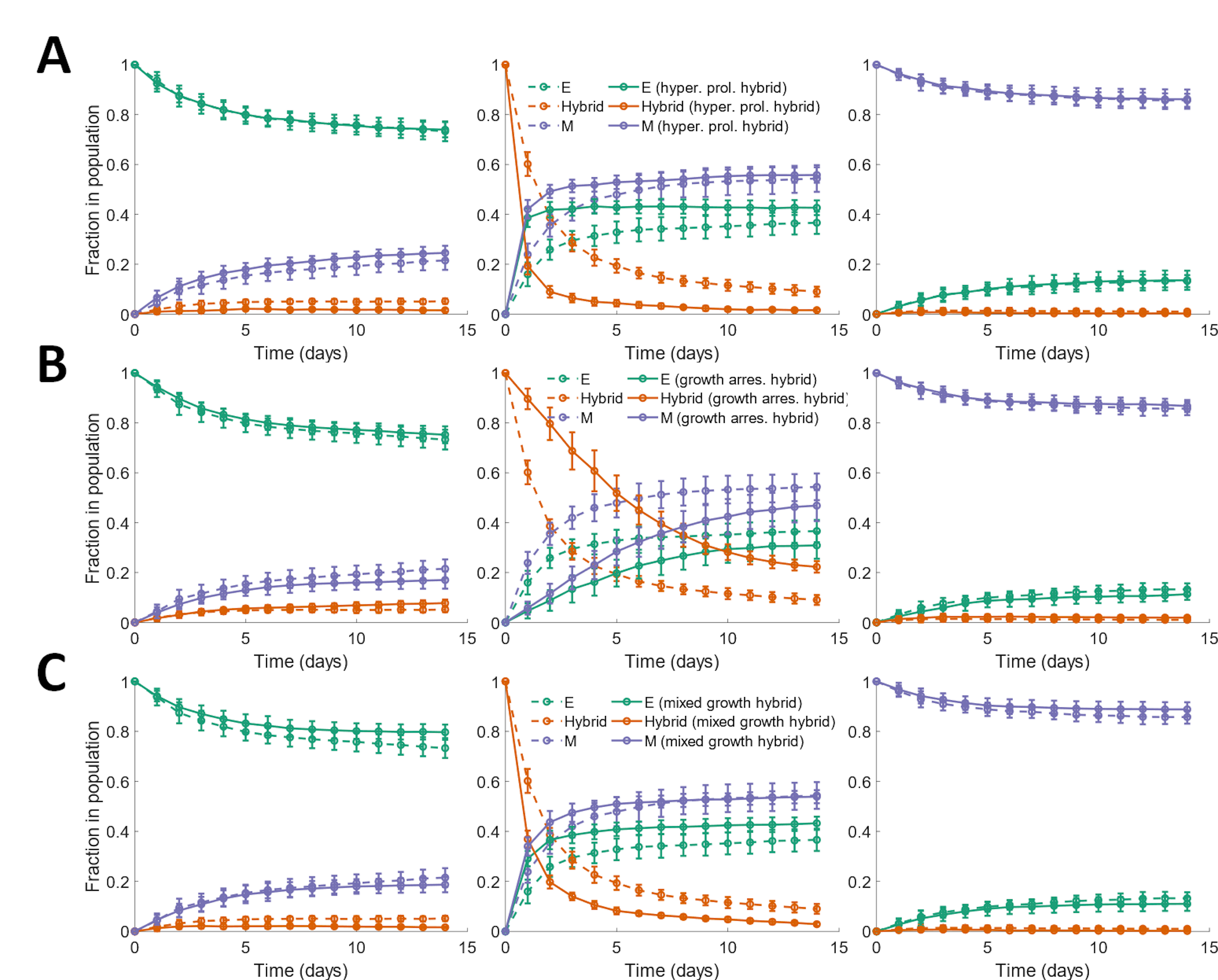

Supplement: S9 Fig — (A) Behavior when hybrid E / M cells exhibit a hyper proliferative phenotype (average doubling time of 8.0 hours). (B) Behavior when hybrid E / M cells exhibit a growth arrested phenotype (average doubling time of 192.0 hours or 8 days). (C) Behavior when hybrid E / M cells exhibit mixed growth phenotype—each hybrid E / M cell was either hyper proliferative (average doubling time of 8.0 hours) or growth arrested (average doubling time of 192.0 hours or 8 days), with equal probabilities. Dashed curves in each plot indicate the behavior when all cells (epithelial, mesenchymal, or hybrid E / M) have the same growth rates (average doubling time of 38.0 hours). In each simulation, η = 2.5×10^4^ with asymmetric partitioning of miR-34a among the daughter cells during hybrid E / M cell division. All results were obtained by averaging over 16 distinct simulation runs. Error bars indicate the standard deviation calculated over these runs. As expected, behavior of epithelial and mesenchymal populations is unaffected by the growth phenotype of hybrid E / M cells within the time period for which the population dynamics were simulated (left and right panels in (A), (B), and (C)). When hybrid E / M cells were hyper-proliferative, the rate of decline in the fraction of these cells in a population that was purely hybrid E / M on day 0 increased ((A); center panel). In contrast, the rate of decline was lowered when hybrid E / M cells exhibited a growth arrested phenotype ((B); center panel). This behavior is a consequence of the high probability of generation of a non-hybrid E / M daughter cell when a hybrid E / M cell divides (Fig 2(A); center panel). Higher growth rate of hybrid E / M cells would lead to epithelial or mesenchymal daughter cells being generated at a faster rate at the expense of hybrid E / M parent cells, resulting in a faster decrease in the fraction of hybrid E / M cells in the population. This decrease is slow when hybrid E / M cells divide at a slower rate ((B); c [file pcbi.1007619.s010.tif]

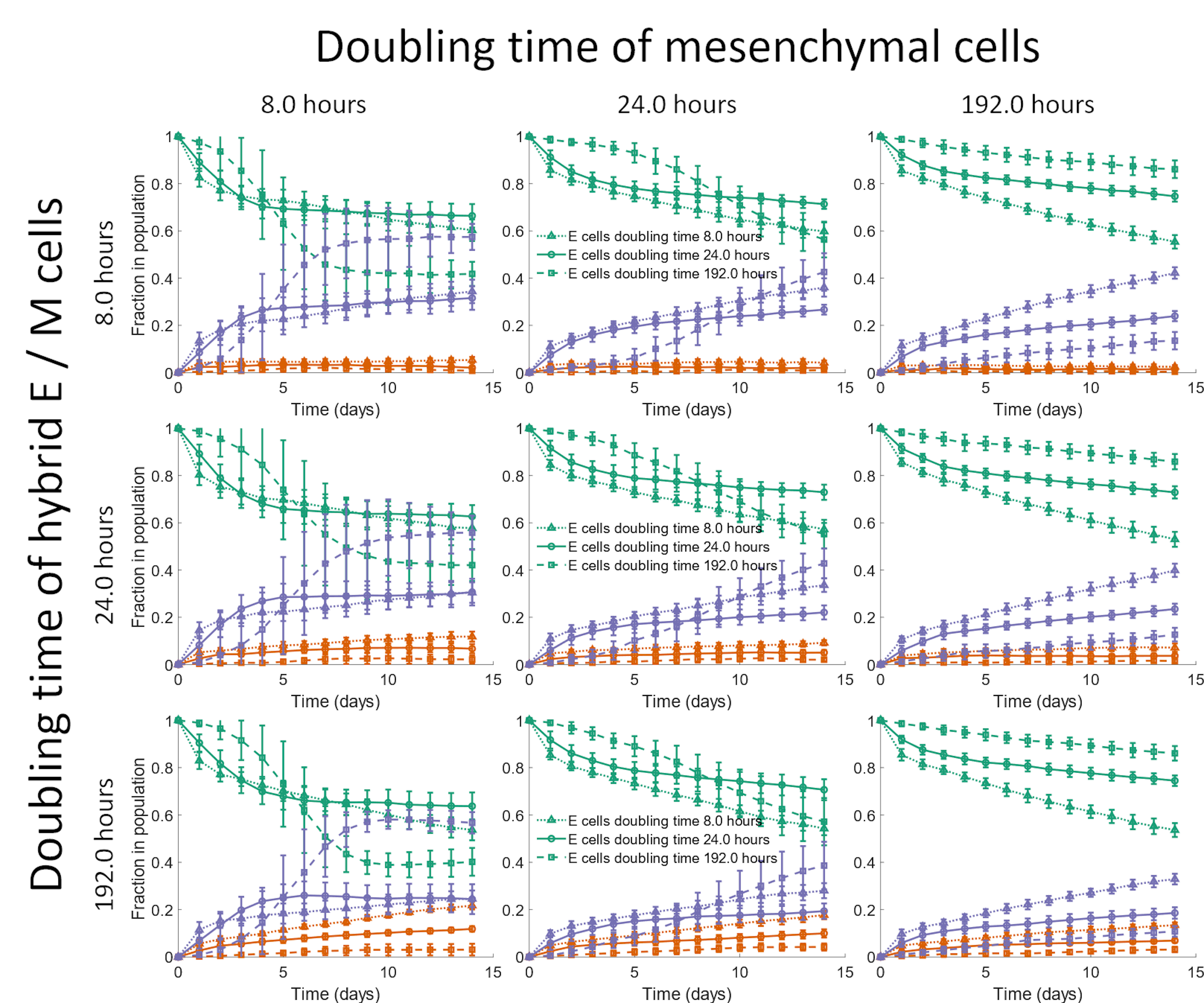

Supplement: S10 Fig — In each panel, the color indicates the fraction in population of a given phenotype—epithelial (green), hybrid E / M (orange), and mesenchymal (purple) while the line type indicates the behavior for different growth rates of epithelial cells—8.0 hours (dotted with triangular markers), 24.0 hours (solid with circular markers), and 192.0 hours (dashed with square markers). In each simulation, η = 2.5×10^4^ with asymmetric partitioning of miR-34a among the daughter cells during hybrid E / M cell division. All results were obtained by averaging over 16 distinct simulation runs. Error bars indicate the standard deviation calculated over these runs. (TIF) [file pcbi.1007619.s011.tif]

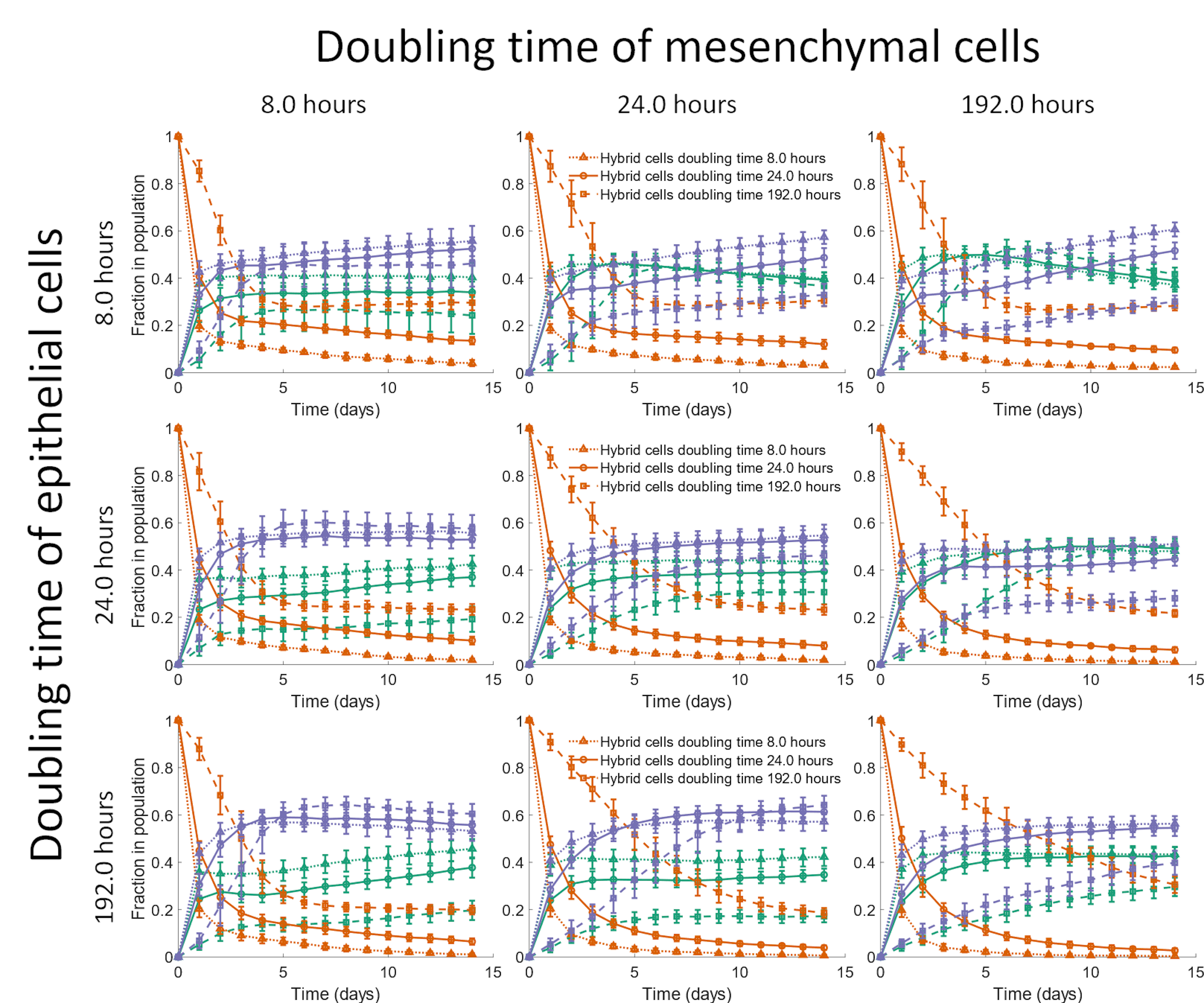

Supplement: S11 Fig — In each panel, the color indicates the fraction in population of a given phenotype—epithelial (green), hybrid E / M (orange), and mesenchymal (purple) while the line type indicates the behavior for different growth rates of epithelial cells—8.0 hours (dotted with triangular markers), 24.0 hours (solid with circular markers), and 192.0 hours (dashed with square markers). In each simulation, η = 2.5×10^4^ with asymmetric partitioning of miR-34a among the daughter cells during hybrid E / M cell division. All results were obtained by averaging over 16 distinct simulation runs. Error bars indicate the standard deviation calculated over these runs. (TIF) [file pcbi.1007619.s012.tif]

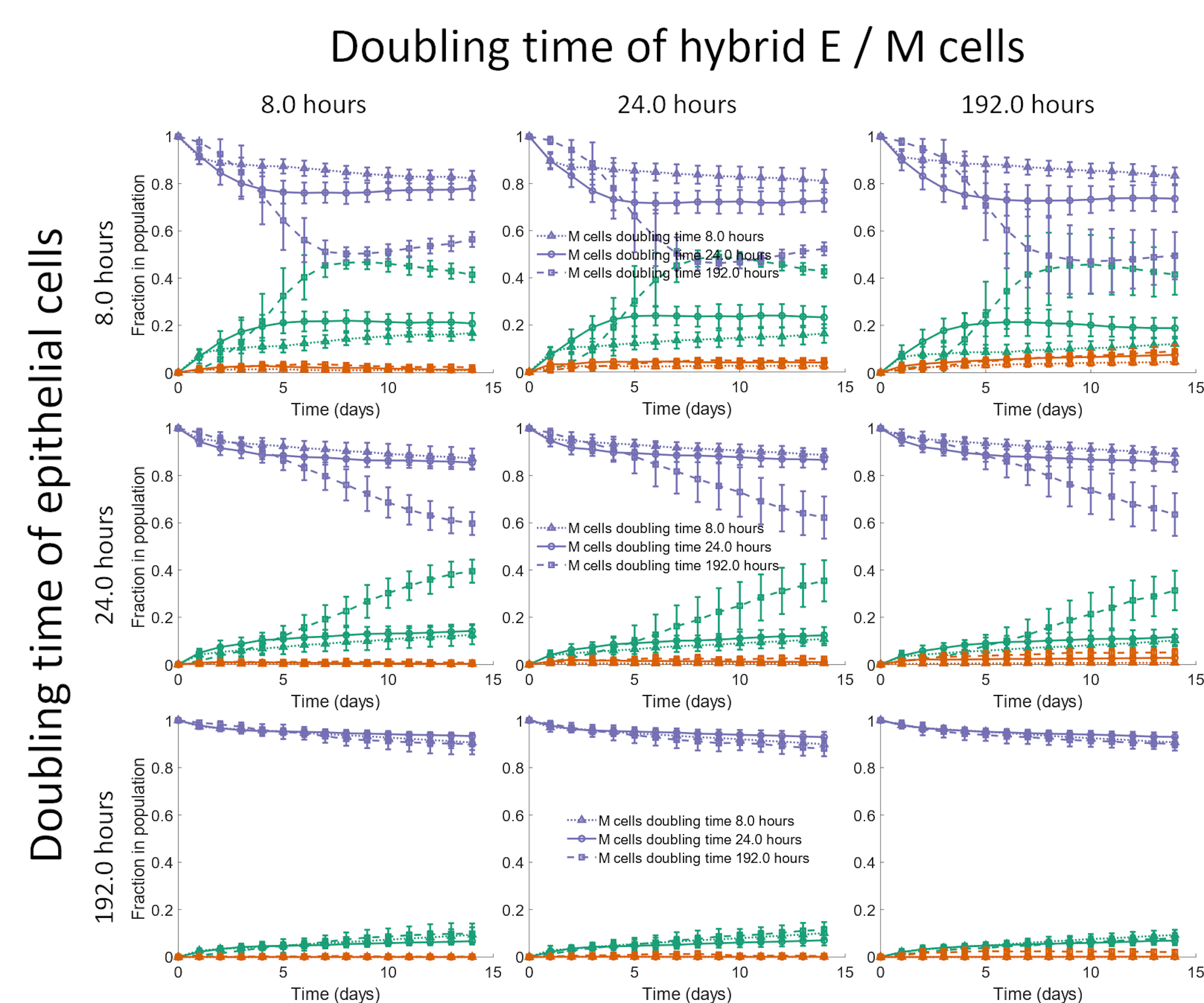

Supplement: S12 Fig — In each panel, the color indicates the fraction in population of a given phenotype—epithelial (green), hybrid E / M (orange), and mesenchymal (purple) while the line type indicates the behavior for different growth rates of epithelial cells—8.0 hours (dotted with triangular markers), 24.0 hours (solid with circular markers), and 192.0 hours (dashed with square markers). In each simulation, η = 2.5×10^4^ with asymmetric partitioning of miR-34a among the daughter cells during hybrid E / M cell division. All results were obtained by averaging over 16 distinct simulation runs. Error bars indicate the standard deviation calculated over these runs. (TIF) [file pcbi.1007619.s013.tif]

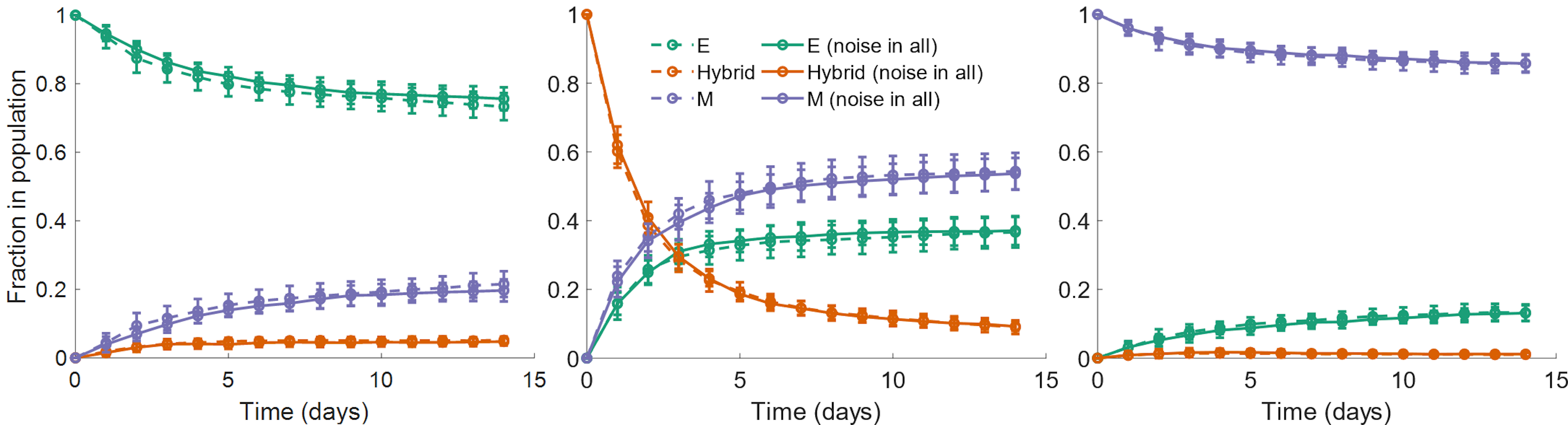

Supplement: S13 Fig — Solid curves indicate the fractions of different phenotypes when noise in the partitioning of all network species is incorporated into the model (CV = 0.25 for all species other than Isig). Dashed curves indicate the behavior when the model only includes noise in the partitioning of Isig. In each simulation, η = 2.5×10^4^ (here, this noise parameter only determines the noise in the partitioning of Isig). miR-34a was asymmetrically partitioned among the daughter cells during hybrid E / M cell division. All results were obtained by averaging over 16 distinct simulation runs. Error bars indicate the standard deviation calculated over these runs. (TIF) [file pcbi.1007619.s014.tif]

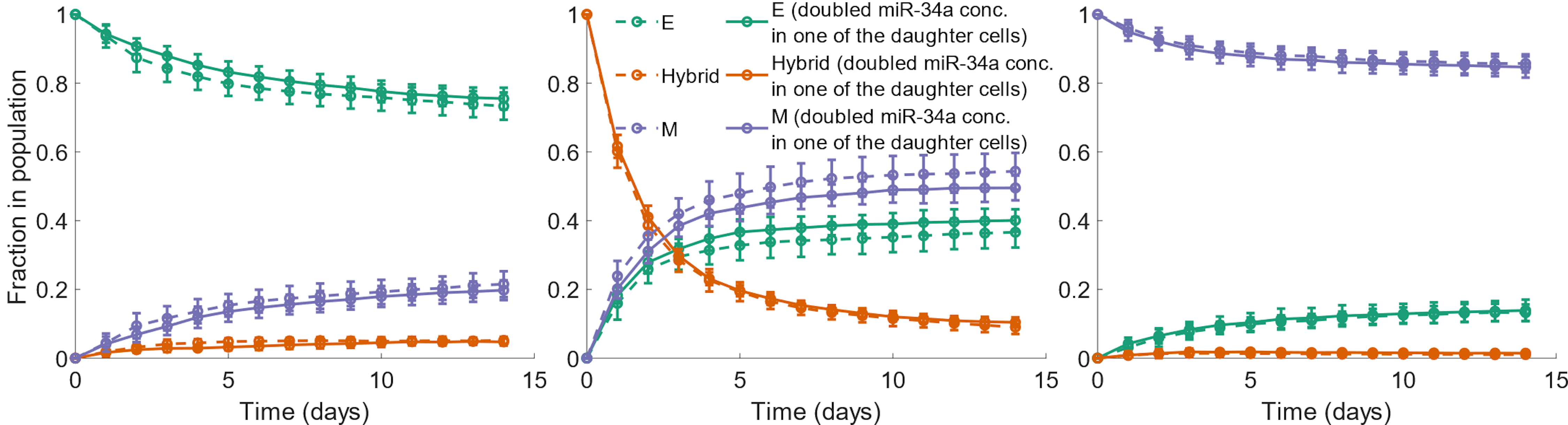

Supplement: S14 Fig — Solid curves indicate the fractions of different phenotypes when, during hybrid E / M cell division, one daughter cells gets no miR-34a while the other gets twice the concentration of miR-34a in the parent cell. Here, the assumption is that during hybrid E / M cell division, all of parent cell miR-34a (note that miR-34a copy number in the parent cell gets approximately doubled right before the parent cell divides) is actively deposited into one of the daughter cells. Dashed curves indicate the behavior when during hybrid E / M cell division, the concentration of miR-34a in one of the daughter cells is set to zero while the concentration of miR-34a in the other daughter cell is kept the same as the concentration of miR-34a in the parent cell. In this case, the assumption is that while parent cell miR-34a is equally divided among the daughter cells during the division of a hybrid E / M cell, in one of the daughter cells, an active process rapidly degrades miR-34a and brings its concentration to zero. In each simulation, η = 2.5×10^4^. All results were obtained by averaging over 16 distinct simulation runs. Error bars indicate the standard deviation calculated over these runs. (TIF) [file pcbi.1007619.s015.tif]

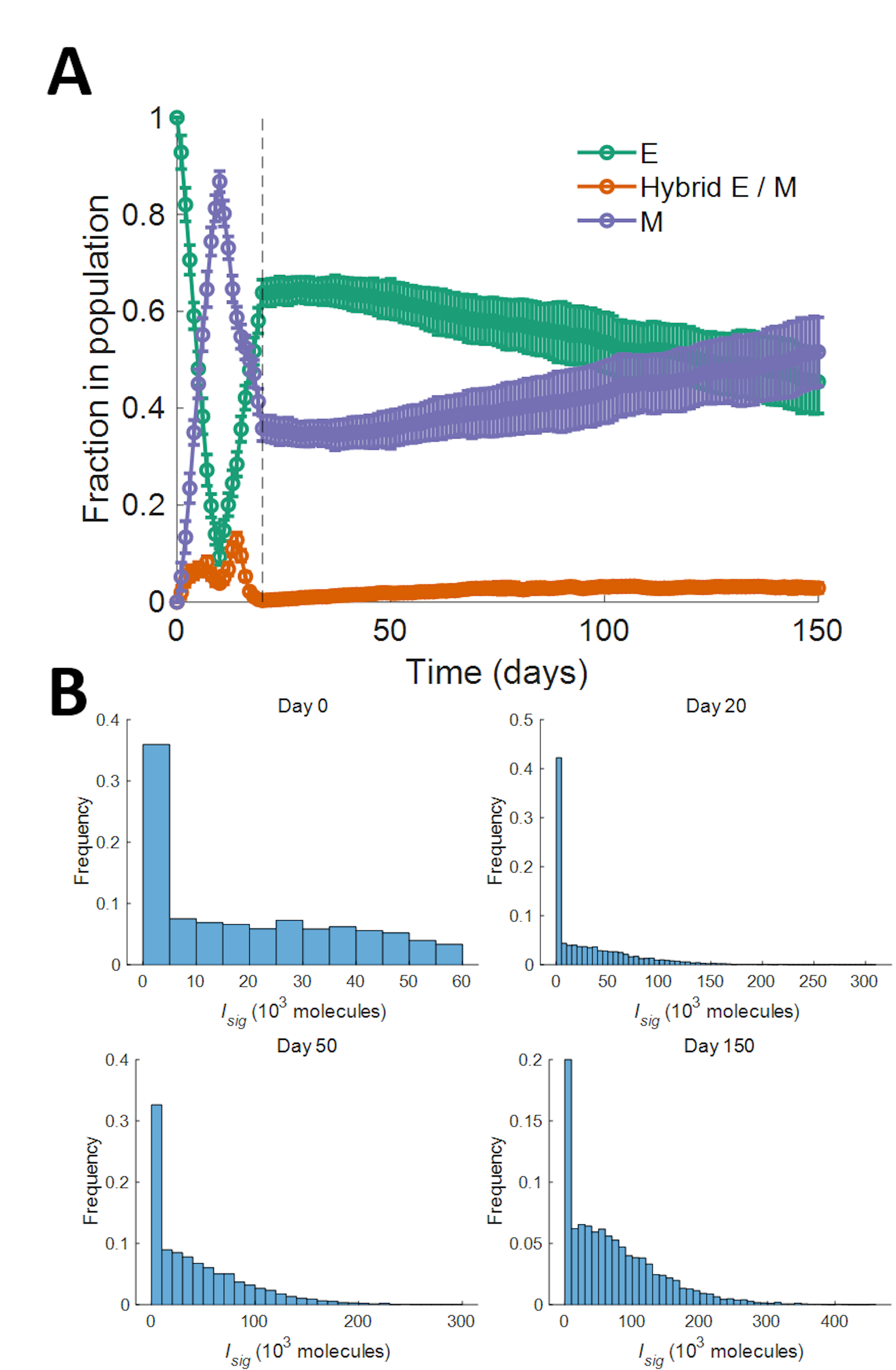

Supplement: S15 Fig — (A) Fraction of epithelial, mesenchymal, and hybrid E / M cells shown over a period of 150 days in the population probed in Fig 4. The black dashed line indicates day 20 after which no addition or subtraction of Isig dosages was administered. Results shown were obtained by averaging over 16 distinct simulation runs. Error bars indicate the standard deviation calculated over 16 independent runs. (B) Distribution of Isig concentration in cells in the population at different time points during the simulation run. Here, η = 2.7×10^4^ and the average doubling time of cells was 38.0 hours. As shown in (B), at the end of the 20-day period, most cells in the population either have Isig concentration close to 0.0 or very high Isig concentration. Few cells have Isig concentration in the region of tri-stable dynamics of the EMP regulatory circuit (Fig 1(B)). Since cells far away from the tri-stable region, which form the bulk of the population on day 20, are highly unlikely to generate a daughter cell with a phenotype different from that of the parent cell, the fractions of cells of different phenotypes in the population do not change much for nearly a month after day 20 when the external addition or withdrawal of Isig dosages was stopped. The population takes a long time to recover from the clustering of Isig concentrations away from the tri-stable region. However, noise in the partitioning of Isig among the daughter cells during cell division eventually drives the Isig concentration in cells back into the tri-stable region and around day 60, we once again start observing the typical dynamics—decrease in the fraction of epithelial cells and increase in the fraction of mesenchymal cells in the population. A small sub-population of hybrid E / M cells is continuously maintained. (TIF) [file pcbi.1007619.s016.tif]

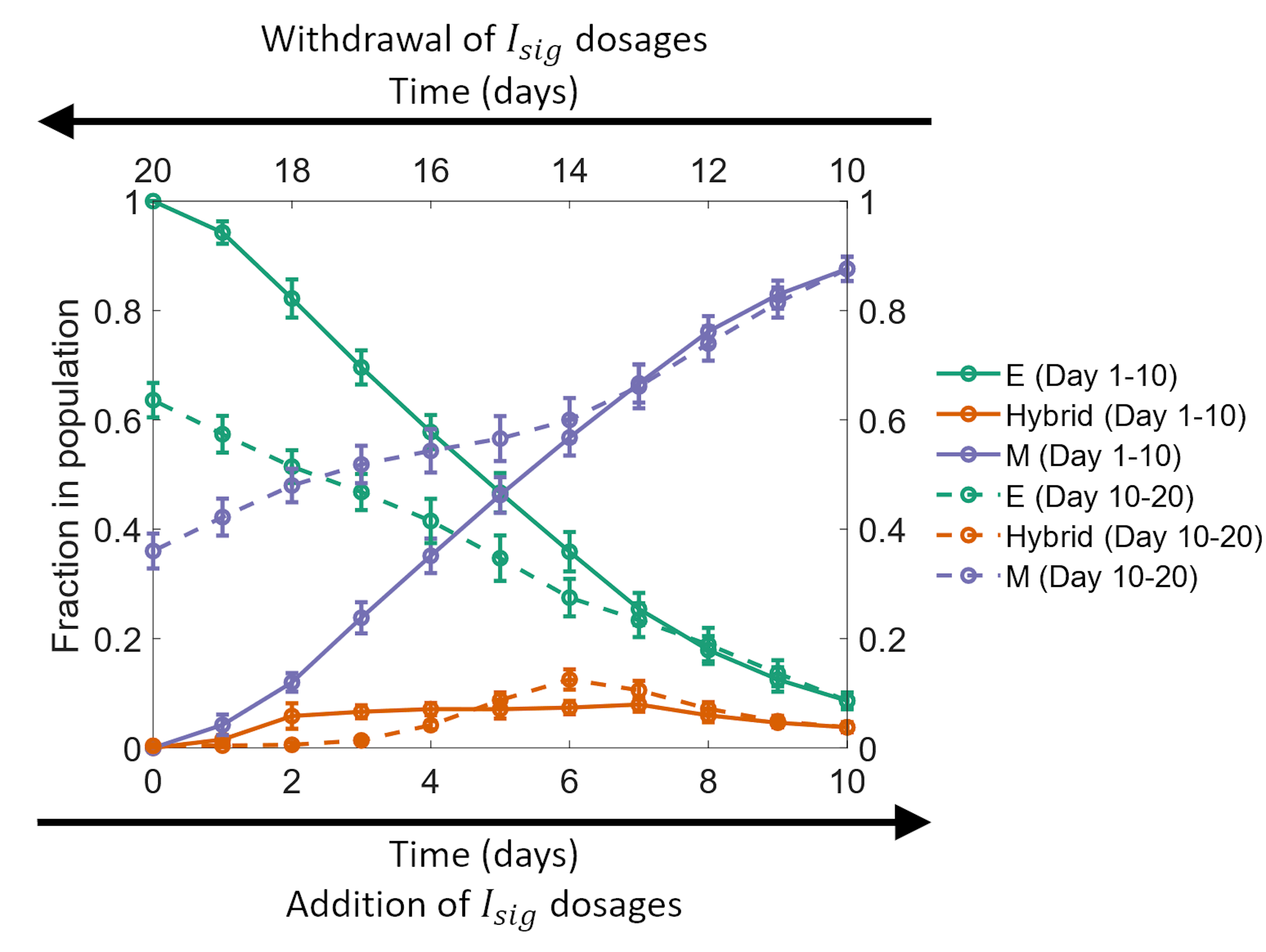

Supplement: S16 Fig — Here, η = 2.5×10^4^ which had given the best fit to experimental data from Ruscetti et al. when incorporating asymmetric distribution of miR-34a among the daughter cells during hybrid E / M cell division into our model. (TIF) [file pcbi.1007619.s017.tif]

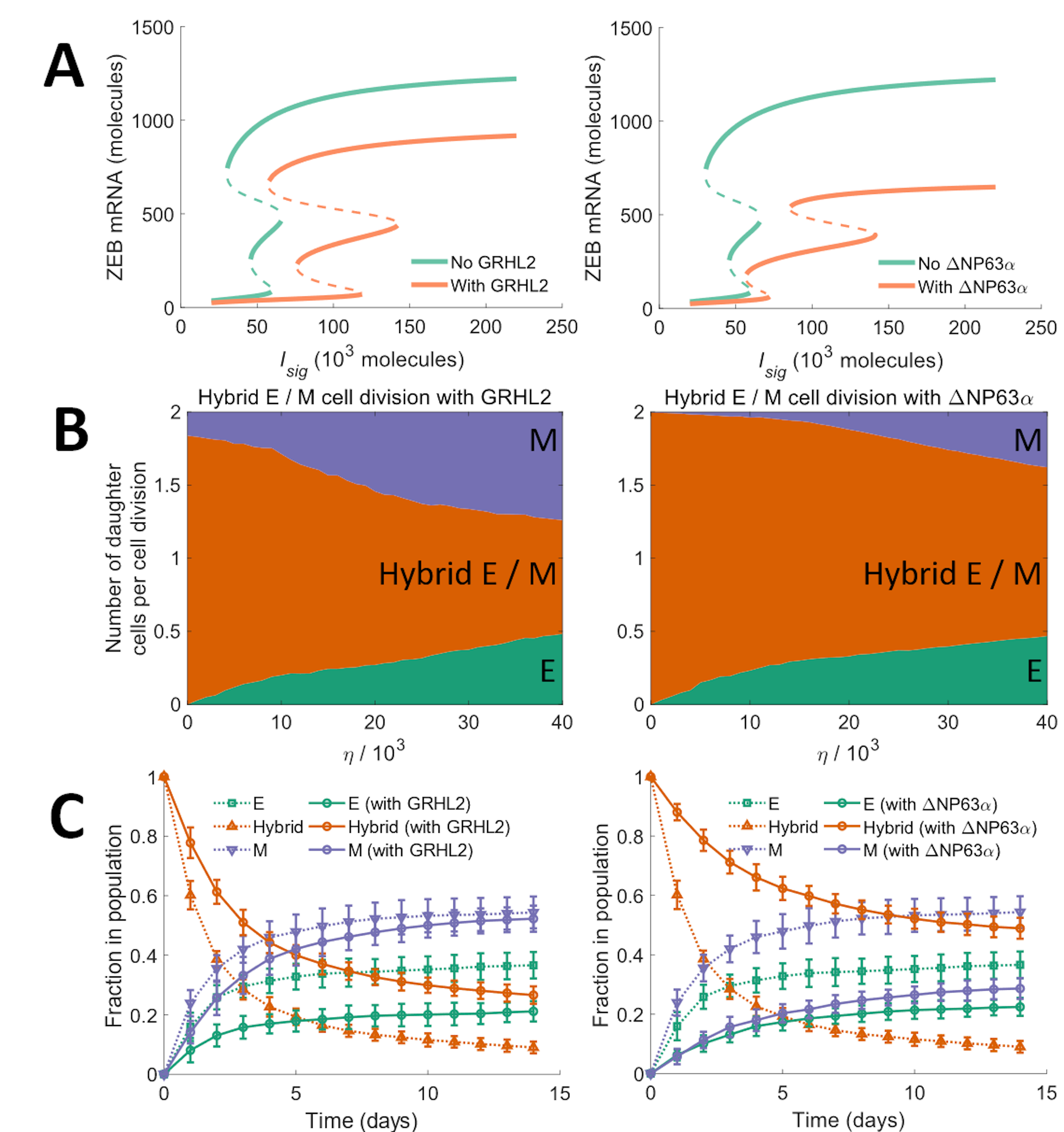

Supplement: S17 Fig — Here, in (B) and (C), η = 2.5×10^4^ which had given the best fit to experimental data from Ruscetti et al. when incorporating asymmetric distribution of miR-34a among the daughter cells during hybrid E / M cell division into our model. (TIF) [file pcbi.1007619.s018.tif]

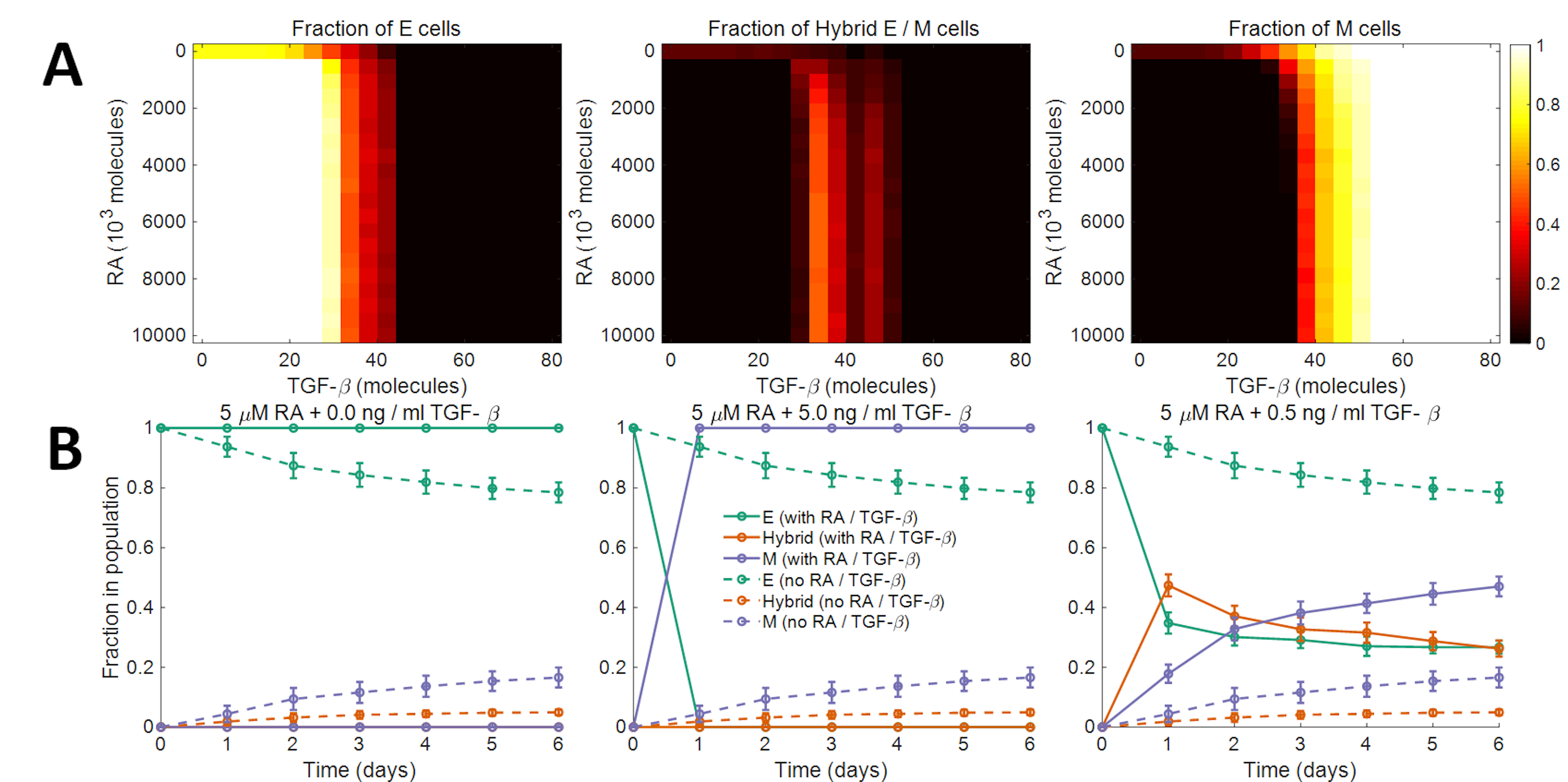

Supplement: S18 Fig — Here, η = 2.5×10^4^ which had given the best fit to experimental data from Ruscetti et al. when incorporating asymmetric distribution of miR-34a among the daughter cells during hybrid E / M cell division into our model. (TIF) [file pcbi.1007619.s019.tif]

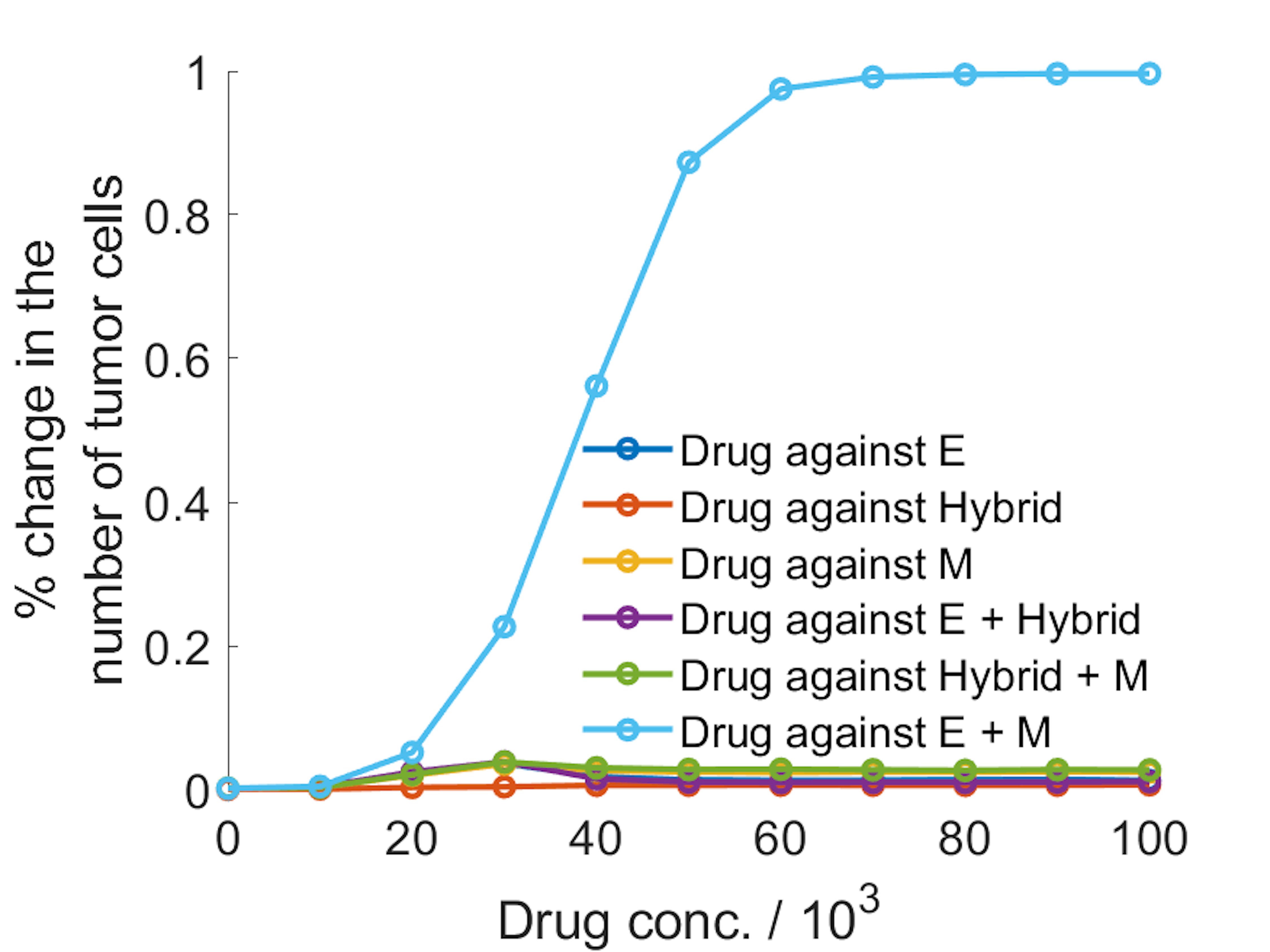

Supplement: S19 Fig — Here, η = 2.5×10^4^ which had given the best fit to experimental data from Ruscetti et al. when incorporating asymmetric distribution of miR-34a among the daughter cells during hybrid E / M cell division into our model. (TIF) [file pcbi.1007619.s020.tif]

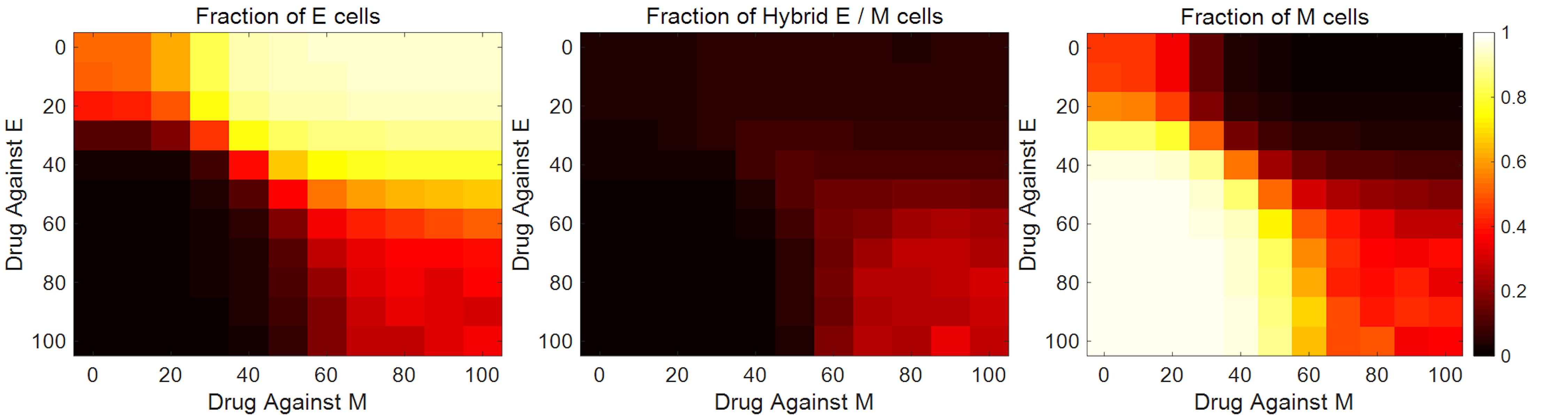

Supplement: S20 Fig — Here, η = 2.5×10^4^ which had given the best fit to experimental data from Ruscetti et al. when incorporating asymmetric distribution of miR-34a among the daughter cells during hybrid E / M cell division into our model. (TIF) [file pcbi.1007619.s021.tif]
